# Structure of infective Getah virus at 2.8 Å resolution determined by cryo-electron microscopy

**DOI:** 10.1038/s41421-022-00374-6

**Published:** 2022-02-11

**Authors:** Aojie Wang, Feng Zhou, Congcong Liu, Dongsheng Gao, Ruxi Qi, Yiheng Yin, Sheng Liu, Yuanzhu Gao, Lutang Fu, Yinhe Xia, Yawei Xu, Chuanqing Wang, Zheng Liu

**Affiliations:** 1 Cryo-electron Microscopy Center, Southern University of Science and Technology, Shenzhen, Guangdong China; 2 College of Animal Science and Veterinary Medicine, Henan Agricultural University, Zhengzhou, Henan China; 3 Henan Dairy Herd Improvement Center, Zhengzhou, Henan China; 4 Institute for Hepatology, National Clinical Research Center for Infectious Disease, Shenzhen Third People’s Hospital, Shenzhen, Guangdong China; 5 Department of Cardiology, Shanghai Tenth People’s Hospital, and Pan-Vascular Research Institute, Heart, Lung, and Blood Center, Tongji University School of Medicine, Shanghai, China

**Keywords:** Cryoelectron microscopy, Molecular modelling

## Abstract

Getah virus (GETV), a member of the genus *alphavirus*, is a mosquito-borne pathogen that can cause pyrexia and reproductive losses in animals. Although antibodies to GETV have been found in over 10% of healthy people, there are no reports of clinical symptoms associated with GETV. The biological and pathological properties of GETV are largely unknown and antiviral or vaccine treatments against GETV are still unavailable due to a lack of knowledge of the structure of the GETV virion. Here, we present the structure of infective GETV at a resolution of 2.8 Å with the atomic models of the capsid protein and the envelope glycoproteins E1 and E2. We have identified numerous glycosylation and S-acylation sites in E1 and E2. The surface-exposed glycans indicate a possible impact on viral immune evasion and host cell invasion. The S-acylation sites might be involved in stabilizing the transmembrane assembly of E1 and E2. In addition, a cholesterol and a phospholipid molecule are observed in a transmembrane hydrophobic pocket, together with two more cholesterols surrounding the pocket. The cholesterol and phospholipid stabilize the hydrophobic pocket in the viral envelope membrane. The structural information will assist structure-based antiviral and vaccine screening, design, and optimization.

## Introduction

Getah virus (GETV) is a mosquito-borne arbovirus, and belongs to the Semliki Forest group of the *Alphavirus* genus within the *Togaviridae* family^1^. Alongside GETV, members in the Semliki Forest Group include Chikungunya virus (CHIKV), Semliki Forest virus (SFV), Mayaro virus (MAYV), Una virus (UNAV), Bebaru virus (BEBV), and O’nyong-nyong virus (ONNV). Among them, CHIKV, ONNV, and MAYV have been reported to cause severe and mortally dangerous infectious diseases in human^2–4^.

GETV was first isolated in Malaysia in 1955 from *Culex gelidus* mosquitoes^5^, and was found to have a worldwide distribution. The primary hosts of GETV include pigs, cattle, foxes, and horses. The first outbreak of GETV among racehorses occurred in Japan in 1978 and outbreaks have re-emerged several times since then in Japan and India^6,7^. The infected horses exhibited pyrexia, urticarial rash on various portions of the body, and edema of the hind legs^8^. GETV has been isolated from newborn piglets that died of acute disease and dead fetuses removed from infected sows in Japan^9,10^. The emergence of GETV in China was found in 2017 in swine farms, resulting in the death of the newborn piglets 5–10 days after birth and in pregnant sows exhibiting stillbirths or fetal mummies^11^. GETV was also reported to infect beef cattle^12^, blue foxes^13^, and wild boars^14^. Neutralizing antibodies of GETV have been detected in various vertebrate species, suggesting that domesticated animals can act as reservoir hosts^15^. Serological investigation of GETV revealed that a widespread infection of GETV occurred in farms across China, and GETV was responsible for significant economic damage^11^. Although the pathogenicity of GETV in humans has not yet been identified, seroepidemiologic investigations have shown that some individuals with a febrile illness have significantly higher GETV antibody titers than in healthy people^16^, suggesting an association of GETV with human diseases. Currently, there is no specific antiviral treatment for animals infected with GETV. Furthermore, the physiological, pathogenic, and epidemic properties of GETV are largely unknown. Considering lessons learned from the ongoing global pandemic of coronavirus disease 2019 (COVID-19) that resulted from the outbreak of severe acute respiratory syndrome coronavirus 2 (SARS-CoV-2), GETV presents a potential risk of becoming a zoonosis. Thus, we should plan and prepare for the possibility of GETV transmission to humans.

Like a typical alphavirus, GETV is a lipid-enveloped, positive-sense single-stranded RNA virus^1^. Mature virions of alphaviruses are spherical particles with a diameter of ~70 nm. The 11-kb genome of GETV encodes two polyproteins. Among them, one polyprotein consists of four non-structural proteins (nsP1–nsP4), and another polyprotein is composed of five structural proteins, including the capsid, E3, E2, 6K, and E1 in order from the N-terminal to the C-terminal^17^. The structures of alphaviruses have been well studied by using cryo-electron microscopy (cryo-EM). These include the structures of Barmah Forest virus (BFV), Eastern equine encephalitis viruses (EEEV), Western equine encephalitis viruses (WEEV), Venezuelan equine encephalitis virus (VEEV), Sindbis virus (SINV), CHIKV, and MAYV, ranging from 3.5 to 13 Å in resolution; there are also structures of CHIKV with its receptor MXRA8^18–26^. These structures reveal a typical architecture for alphavirus organization: viral RNA occupies the center of the particle and extends to ~140 Å radially, and the RNA is surrounded by the capsid protein shell ranging between 140 and 200 Å radially; the lipid membrane shell (200–255 Å radially) separates the capsid and the outer glycoprotein shell, and the outer shell and spikes protruding outward (255–350 Å radially) are formed by the E1 and E2 glycoproteins. In addition, high-resolution maps have assisted the construction of atomic models for the capsid and the E1 and E2 glycoproteins^20,22,24,25^. Moreover, Mxra8, the receptor of CHIKV, was found to bind into a cleft created by two adjacent CHIKV E1–E2 heterodimers^26^.

In a previous study, we have isolated a new GETV strain, the V1 strain, from pregnant sows that had abortions. We have demonstrated that the V1 strain of GETV had a strong cytopathic effect^27^. In this study, we utilized mouse models to investigate the infectiousness and pathogenicity of mature GETV. To gain structural insight into GETV, we determined the structure of the mature GETV-V1 virion at 2.8 Å resolution by cryo-EM, the highest resolution for an alphavirus to date.

## Results

### GETV is a mosquito-borne arbovirus

GETV appears to be maintained in a natural cycle between mosquitoes and various vertebrate hosts^28^. There is also suspicion that GETV can be transmitted directly between horses, likely via aerosols or direct contact^8^. There is no clear evidence concerning how GETV is transmitted by mosquitoes. Therefore, we first investigated the transmission and infectiousness of GETV using a mouse model (Supplementary Fig. S1). As shown in Supplementary Fig. S1a, mice (2-day old) were inoculated oronasally with 10 μL (10^6^ TCID_50_/mL, 50% tissue culture infectious dose) of the GETV-V1 strain, and were then housed in a cage with their GETV-free littermates. Total RNA was extracted from tissues, including spleen, lung, cerebral cortex, and various lymph nodes at 5 days postinoculation (DPI), and subjected to RT-PCR for GETV detection (Supplementary Fig. S1g, h). The uninfected mice remained negative for GETV (Supplementary Fig. S1a). When male adult inoculated mice (2-month old) were housed with their uninfected male littermates (Supplementary Fig. S1b), all mice tested GETV-positive at 5 DPI. Multiple wounds were found in various parts of their body, indicating that the transmission of GETV occurred through scratching and biting.

We also conducted experiments based on a special device we designed to connect two cages via a pipe. We placed screens at the two ends of the pipe to separate the mice and to allow mosquitoes to fly between the cages freely (Supplementary Fig. S1c). In a control test shown in Supplementary Fig. S1d, GETV-free mice in the two cages lived with mosquitoes and tested negative after 5 days, indicating that the mosquitoes used in our study were GETV-free. Mice were oronasally inoculated with GETV, and then placed with their noninoculated littermates in the left-hand cage. Additional GETV-free littermates were placed in the right-hand cage, and mosquitoes were introduced into the device (Supplementary Fig. S1e). At 5 DPI, all of the mice in the left-hand cage were GETV-positive, and 15% of the mice in the right-hand cage were also positive. In another control test, no mosquitoes were introduced, all of the noninoculated mice in the right-hand cage remained negative after five days (Supplementary Fig. S1f). These results confirmed that GETV is a mosquito-borne arbovirus, not an airborne virus. GETV was mainly spread by mosquito biting, traveling in the blood between mice and mosquitos. GETV can also transmit between mouses, most likely through direct contact with blood and body fluids.

### Pathogenicity of the GETV-V1 strain

GETV has been reported to be associated with reproductive losses in pregnant sows^11^. In the present study, we investigated the pathogenicity of GETV in pregnant mice. Mice were inoculated oronasally with 100 μL (10^6^ TCID_50_/mL) of the GETV-V1 strain in early- (embryonic day 6, E6), middle- (E10), and late-gestation (E14). The inoculated E6 group mice displayed severe abortion (Supplementary Fig. S2a) and delivered fetal mummies (Supplementary Fig. S2b, f). The E10 group delivered mainly mummies or stillbirths but also delivered a small proportion of healthy pups (Supplementary Fig. S2a–f). The E14 mice delivered a mixture of mummies, stillbirths, and live infants, although some newborn mice were weak (Supplementary Fig. S2a–f). In contrast, no birth defects were detected in the mice inoculated with DMEM at E6 (Supplementary Fig. S2e, f). Surprisingly, although GETV-infected pregnant mice displayed serious reproductive losses, no other disease symptom was discovered; both miscarriage and maternity mice appeared normal. These results suggested that placental and fetal infection occurs when pregnant mice are infected with GETV, eventually causing fetal damage, such as abortion, mummies, or stillbirths.

**Fig. 1:**
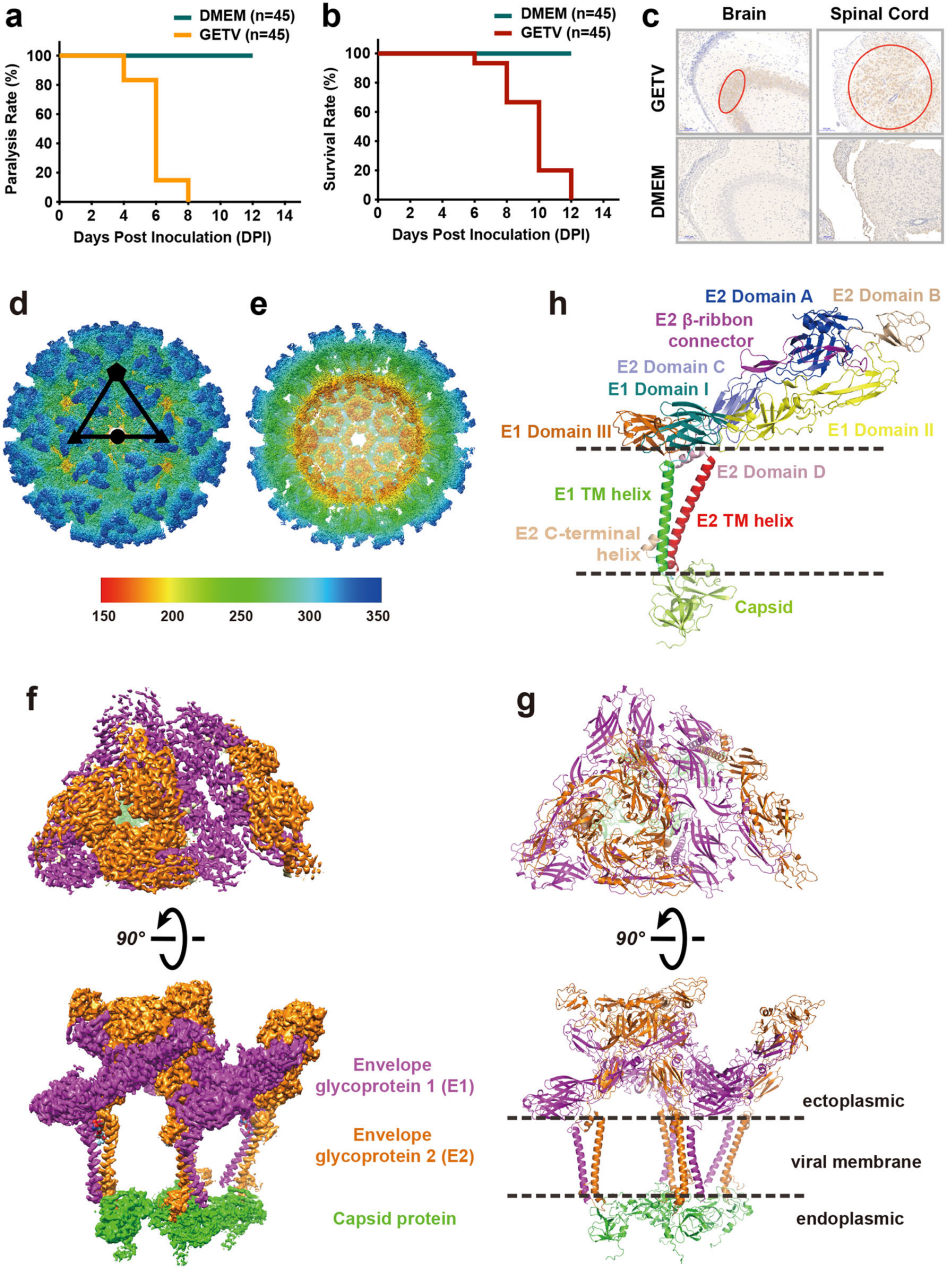
Overall structure of the infectious GETV virion determined by cryo-EM at 2.8 Å resolution. **a**–**c** Ninety 3-day-old mice were randomly divided into two groups and inoculated oronasally with strain GETV-V1 suspension or an equal amount of DMEM. **a** Motor disabilities displayed in GETV-inoculated mice at 4 DPI; by 8 DPI, all of the GETV-inoculated mice displayed paralysis. **b** Mice inoculated with GETV died at 6 DPI, and all of the inoculated mice were dead by 12 DPI. **c** Tissue from hippocampal dentate gyrus in the brain and spinal cord under lumbar vertebrae were subjected for immuno-histochemistry analysis. There were abundant GETV antigens distributed in the brain and spinal cord under lumbar vertebrae in the inoculated newborn mice (highlighted in red circles). **d**–**h** Cryo-EM structure of GETV at 2.8 Å resolution. **d** GETV virion showing the external surface with assigned symmetry axes. **e** Central cross-section of the GETV density map. **f**, **g** Density map and atomic model of one asymmetric unit. The E1, E2, and capsid proteins are colored separately in magenta, orange and green. **h** Atomic model of E1–E2–capsid heterotrimer. Different subdomains of E1 (Domain I, II, III, and TM helix) and E2 (Domain A, B, C, D, TM helix, and C-terminal helix) are shown, following the previous definition for alphaviruses^33,34^.

Next, we investigated the pathogenicity of GETV in newborn mice. 3-day-old mice were inoculated oronasally with 10 μL (10^6^ TCID_50_/mL) of the GETV-V1 strain, and clinical signs were observed daily. At 4 DPI, the inoculated mice displayed mobility impairments in pelvic limbs (Fig. 1a and Supplementary Video S1). By 8 DPI, all inoculated mice displayed paralysis, mainly in pelvic limbs. Nearly 20% of inoculated mice died at 6 DPI (2 days after their paralysis manifestation), and all of the inoculated mice died by 12 DPI (Fig. 1b). On the contrary, no mice exhibited paralysis or death in the control group (Fig. 1a, b). Immunohistochemical staining revealed that abundant GETV antigens were distributed in the hippocampal dentate gyrus of the brain and in the spinal cord under lumbar vertebrae in the inoculated newborn mice (Fig. 1c). Interestingly, no such clinical signs were observed when 7-day-old newborn and 2-month-old adult mice were inoculated with GETV, and GETV was not detected in the brain or spinal cord (Supplementary Fig. S3). This suggests that GETV is only transmitted into the brain before the blood-brain barrier is established^29^, and that the mobility impairments in pelvic limbs are possibly caused by viral infection in the nervous system.

### Cryo-EM structure of GETV

To gain structural insight into GETV, we determined the structure of the infective GETV virion by cryo-EM. Virion particles were purified and characterized by polyacrylamide gel electrophoresis (Supplementary Fig. S4), by mass spectrometry (Supplementary Figs. S5 and S6), by negative-stain electron microscopy (Supplementary Fig. S7), and cryo-EM (Supplementary Fig. S8). The whole structure of GETV was determined at 4.1 Å resolution using Relion3.1 and JSPR softwares (Supplementary Fig. S9). To overcome the heterogeneity of the ~70 nm virus, a block-based reconstruction method was adopted (Supplementary Figs. S9 and S10)^30,31^. Three blocks (5/3/2-fold symmetry axis) of GETV cryo-EM maps at 2.81/2.92/2.85 Å were acquired, respectively (Supplementary Figs. S10, S11 and Table S1). The density maps of the capsid in the pentamers were sufficient to observe the bulk residues. However, the densities for the capsid hexamers were still intermittent. The quality of this map was improved upon the averaging of 15 equivalent capsid densities from the three and twofold symmetry axis blocks. After averaging, the correlation coefficient between the densities of pentamer and hexamer was 0.92 (Supplementary Fig. S12). By combining these three high-resolution densities, the overall structure of GETV was obtained (Fig. 1d, e). Similar to other alphaviruses, GETV has an icosahedral symmetry (*T* = 4), comprising 60 quasi-threefold symmetric trimers (Q-trimer) and 20 icosahedral threefold symmetric trimers (I-trimer)^22^. There are 240 capsid proteins connected to the corresponding E2 proteins. Each asymmetric unit (ASU, Fig. 1f, g) consists of four E1–E2–capsid heterotrimers (Fig. 1h), with three heterotrimers forming one unabridged Q-trimer connected to one heterotrimers from the I-trimer. Interacting regions between the GETV structural proteins are presented in Supplementary Fig. S13 and listed in Supplementary Table S2.

The atomic model of the E1–E2–capsid heterotrimers includes the full-length sequences of E1 and E2, as well as residues 111–268 of the capsid protein. The density for residues 1–110 of the pentamer capsid was not visible in the cryo-EM map, suggesting a flexible architecture in the core of the virion. Notably, we subjected the sequence to the recently- released AlphaFold 2^32^, and only flexible loop structures were predicted.

The majority of the E1 ectodomain of GETV is divided into 3 subdomains (Fig. 1h): domain I (residues 1–36, 130–164, and 274–281), domain II (residues 37–129 and 165–273; of which residues 86–96 form a hydrophobic fusion loop that inserts into the canyon formed by E2’s A and B subdomains), and domain III (residues 282–379)^33^. Each of E1’s three domains has a secondary structure of antiparallel β strands. The remaining ectodomain amino acids (residues 380-401) comprise the stem-loop, which connects domain III and the transmembrane (TM) helix. The E2 ectodomain comprises three subdomains (Fig. 1h): domain A (residues 1–143), domain B (173–242), and domain C (270–344)^34^. In addition, E2’s residues 144–172 and 243–269 form the β-ribbon.

### Molecular interactions in the E1–E2–capsid heterotrimer

**Fig. 2:**
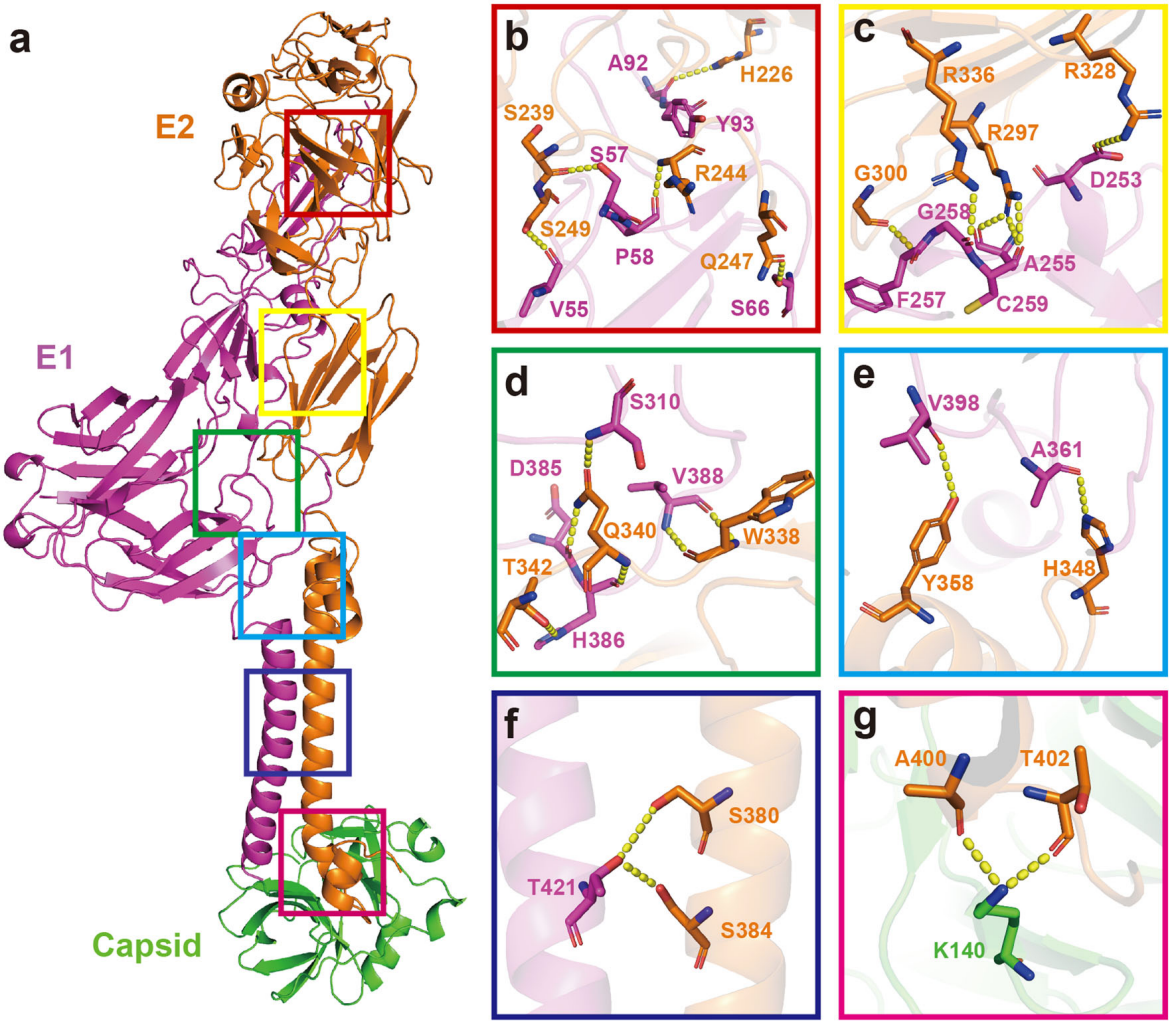
Interaction of GETV protein subdomains in the E1–E2–Capsid heterotrimer. **a** Distribution of interaction regions between E1 and E2 and between E2 and the capsid protein. **b**–**f** Zoomed-in views from **a** of the interaction regions between E1 and E2. **g** Zoomed-in view from **a** of the interaction region between E2 and the capsid protein. The yellow dashed lines indicate the distance between the atoms involved in the interaction (the cut-off distance is 3.5 Å).

The E1, E2, and capsid proteins form the fundamental heterotrimer (Fig. 1h). There are five regions where the E1 and E2 proteins interact (Fig. 2a). In the first region, E1’s V55, S57, P58, and S66 residues interact with the E2’s residues S239, R244, Q247, and S249 (Fig. 2b). This first region is located near the tip of E1’s domain II and the E2’s β-ribbon, which is similar to that of other alphaviruses (e.g., E1 S57 with E2 H164 in SINV^22^; and E1 S57 with E2 H170 in CHIKV^34^). Other residues in this interaction region include E1’s A92 and Y93 and E2’s H226. These three residues are located at the interface between E1’s fusion loop and the E2 domain B, similar to reports for SFV^35^ and CHIKV^34^.

In the second interaction region, E1’s D253, A255, F257, G258, and C259 residues interact with E2’s R297, G300, R328, and R336 residues (Fig. 2c). These interactions occur near the tail of E1’s domain II and E2’s domain C. In the third interaction region — located at E1’s stem-loop, the domain III loop and the tail loop of E2 domain C — E1’s S310, D385, H386, and V388 residues interact with E2’s W338, Q340, and T342 residues (Fig. 2d). Together with the second interaction region, these two areas may be required for E1/E2 dimerization. The E1 and E2 proteins pulled together through these interaction areas formed near the stem-loop of E1.

The fourth interaction region is located at E1’s stem-loop, the domain II loop, and E2 domain D, near the hydrophobic pocket (Fig. 2e). E1’s A361 and V398 residues interact with E2’s H348 and Y358. This region is thought to be functionally important for the stability of a hydrophobic pocket (see subsection below). The fifth interaction region involves E1’s T421 and E2’s S380 and S384 residues (Fig. 2f). This region is located between the E1 and E2 TM helixes and the interactions stabilize these two helixes. There is also an interaction region between E2 and the capsid protein that is responsible for fixing the E1–E2 heterodimer on the capsid. E2’s A400, T402 residues interact with capsid protein’s K140 residue (Fig. 2g). Nearly half of these residues (14/37) described above are conserved in alphaviruses (Supplementary Figs. S14–S16).

### Intra- and Inter-ASU interactions increase virion stability

**Fig. 3:**
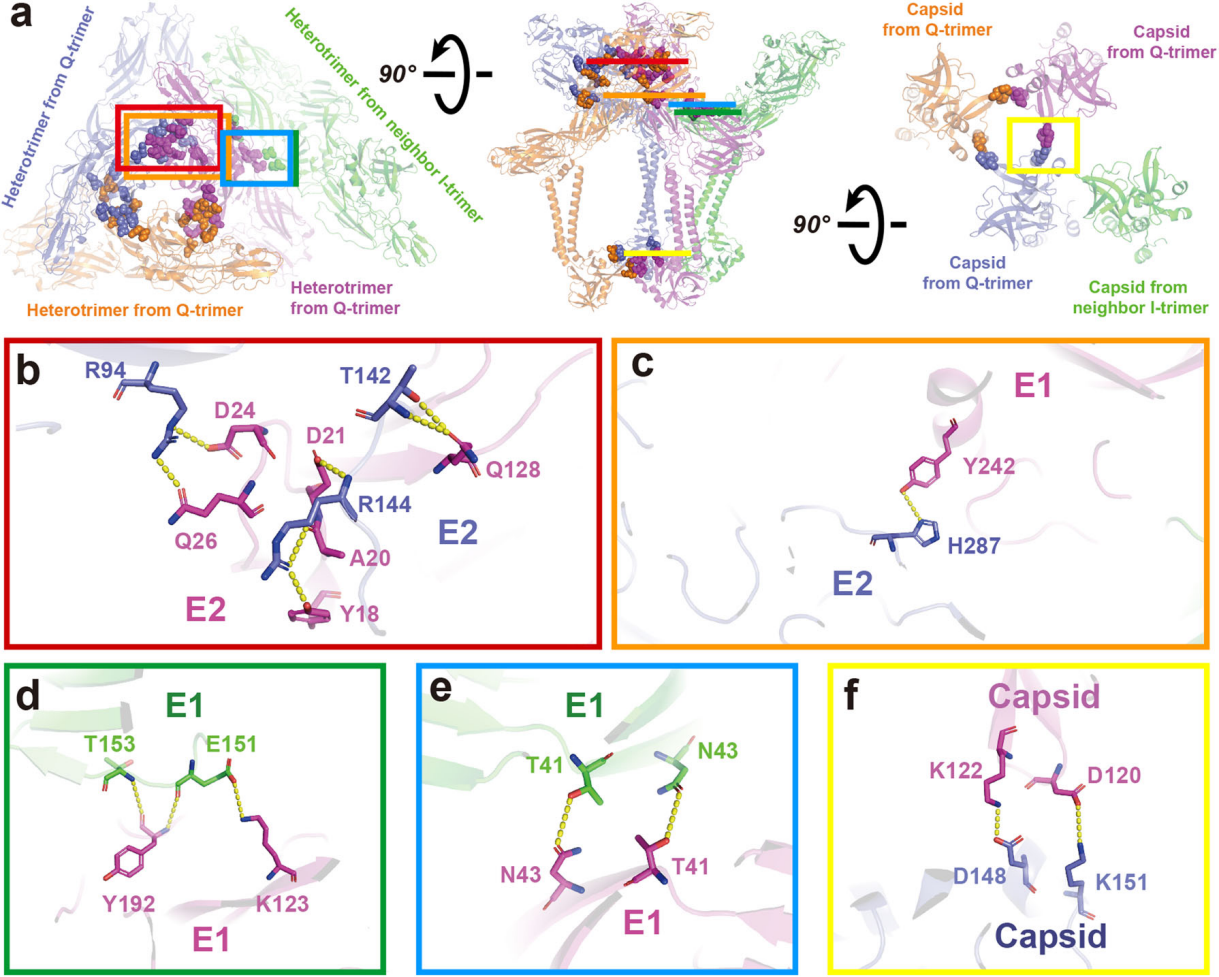
Interaction of GETV protein domains in the ASU. **a** The top, side, and bottom view of the ASU. Four E1–E2–capsid heterotrimers in the ASU are colored slate, magenta, orange, and green. Residues involved in the interaction are displayed as spheres with the same color as the corresponding heterotrimers. The slate, magenta, and orange heterotrimers form the Q-trimer, and the green heterotrimer is from the neighboring I-trimer. **b** Zoomed-in view from the red box in **a** of the interaction between E2 (magenta) and the neighboring E2 (slate) protein. **c** Zoomed-in view from the orange box in **a** of the interaction between E1 and the neighboring E2 protein. **d**, **e** Zoomed-in views from the green and blue boxes in **a**, showing the interactions between E1 (magenta) and the neighboring E1 (green), which also represent the interactions between Q-trimer and I-trimer. **f** The interaction between one capsid protein (magenta) and a neighboring capsid protein (slate) in the Q-trimer. The yellow dashed lines indicate the distance between the atoms involved in the interaction (the cut-off distance is 3.5 Å).

The E1–E2–capsid heterotrimer forms the Q-trimer and I-trimer. The ASU is formed by one Q-trimer and one-third of an I-trimer. Within the Q-trimer, there are three regions for inter-molecular interactions (Fig. 3a–c, f). The E2–E2 homo-dimeric interaction occurs between the middle region of domain A in the first E2 protein and a region of the second E2 protein comprising the joint of domains A and C; this region is positioned near by the tail of the β-ribbon domain (Fig. 3b). The second interaction is mediated by the helix in E1’s domain II and the E2’s domain C to stabilize the Q-trimer (Fig. 3c). Residues K151 and D148 in one capsid interact with D120 and K122 in the neighboring capsid (Fig. 3f). In addition, E1 of the I-trimer interact with the adjacent E1 of the Q-trimer at two regions (Fig. 3d, e). E1’s T153 and E151 in domain I of the I-trimer interact with residues Y192 and K123 at the loop of E1’s domain II in the Q-trimer (Fig. 3d). Residues T41 and N43 located at the β sheets of domain II in two E1s symmetrically interact with each other (Fig. 3e).

**Fig. 4:**
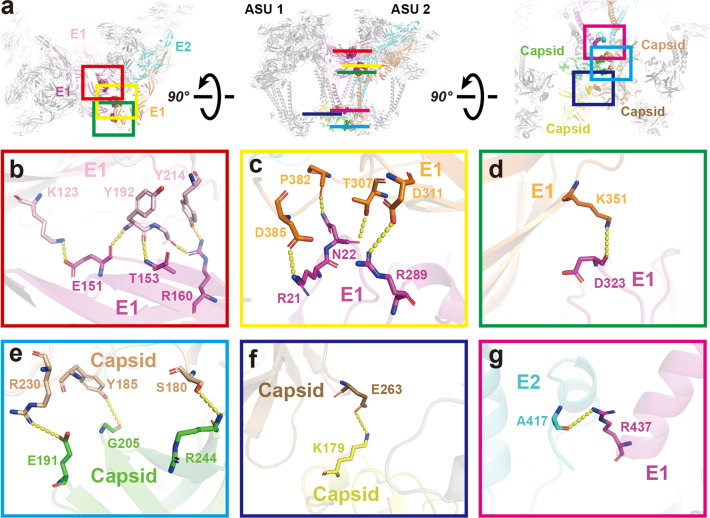
Interactions of GETV protein domains between two ASUs. **a** The top, side, and bottom view of two adjacent Q-trimers. The E1, E2 and capsid proteins participating in the interactions are colored; other proteins are gray. Residues involved in the interaction are displayed as spheres, in the same color as the corresponding proteins. **b**–**d** Zoomed-in views of the interactions between E1 (magenta) of ASU1 and E1 (pink) of the neighboring E1–E2–capsid heterotrimer, along with another E1 (orange) of the heterotrimer in ASU2. **e**, **f** Two sets of capsid–capsid interactions occurring between the contiguous capsids of ASU1 and ASU2. Green and yellow capsids are in the ASU1, and the other two capsids are in the ASU2. **g** Interaction region between E1 (magenta, the same E1 protein in **b**, **c** and **d**) and E2 (cyan, the same E1 heterotrimer in **c** and **d**). The yellow dashed lines indicate the distance between the atoms involved in the interaction.

Six regions form contacts between two ASUs (Fig. 4a). The E1 subunit of the Q-trimer from ASU1 interacts with an adjacent Q-trimer from AUS2 in three regions (Fig. 4b–d). The first interaction region is located between E1’s domain I in ASU1 and E1’s domain II in ASU2. Multiple residues, including K123, Y192, Y214 and E151, T153, and R160 are involved (Fig. 4b). The second interaction region is located between one E1’s domain I (residues R21, N22, and R289) and another E1’s domain III (residues D385, P382, T307, and D311) (Fig. 4c). The third interaction region is located between two E1 proteins, where residues D323 and K351 locate in the E1’s domain III from two adjacent ASUs (Fig. 4d). Residues R437 and A417 are located between the tail of E1 and the C-terminal helix of E2 (Fig. 4g). Except for those interactions between the envelope glycoproteins, two sets of capsid–capsid interactions between two ASUs were also observed (Fig. 4e, f). Interestingly, most of these interacting residue pairs occur between acidic and basic residues. For instance, E151 and K123 (Fig. 4b), R21 and D385, R289 and D311 (Fig. 4c), D323 and K351 (Fig. 4d), E191 and R230 (Fig. 4e), and K179 and E263 (Fig. 4f). Such electrostatic attractions increase the stability of protein–protein interaction.

We summarize all of the protein–protein interactions in Supplementary Table S3, including hydrogen bonds, salt bridges, and van der Waals contacts. There are a total of 47 interactions, among which 28 have been described in earlier studies, and 19 of which are first-identified in the present study. Taken together, the protein–protein interactions of the E1–E2–capsid heterotrimer, within one ASU, and between ASUs stabilize the structural assembly of the GETV, as well as other alphaviruses.

### Glycosylation sites in the E1–E2 ectodomain are surface-exposed

**Fig. 5:**
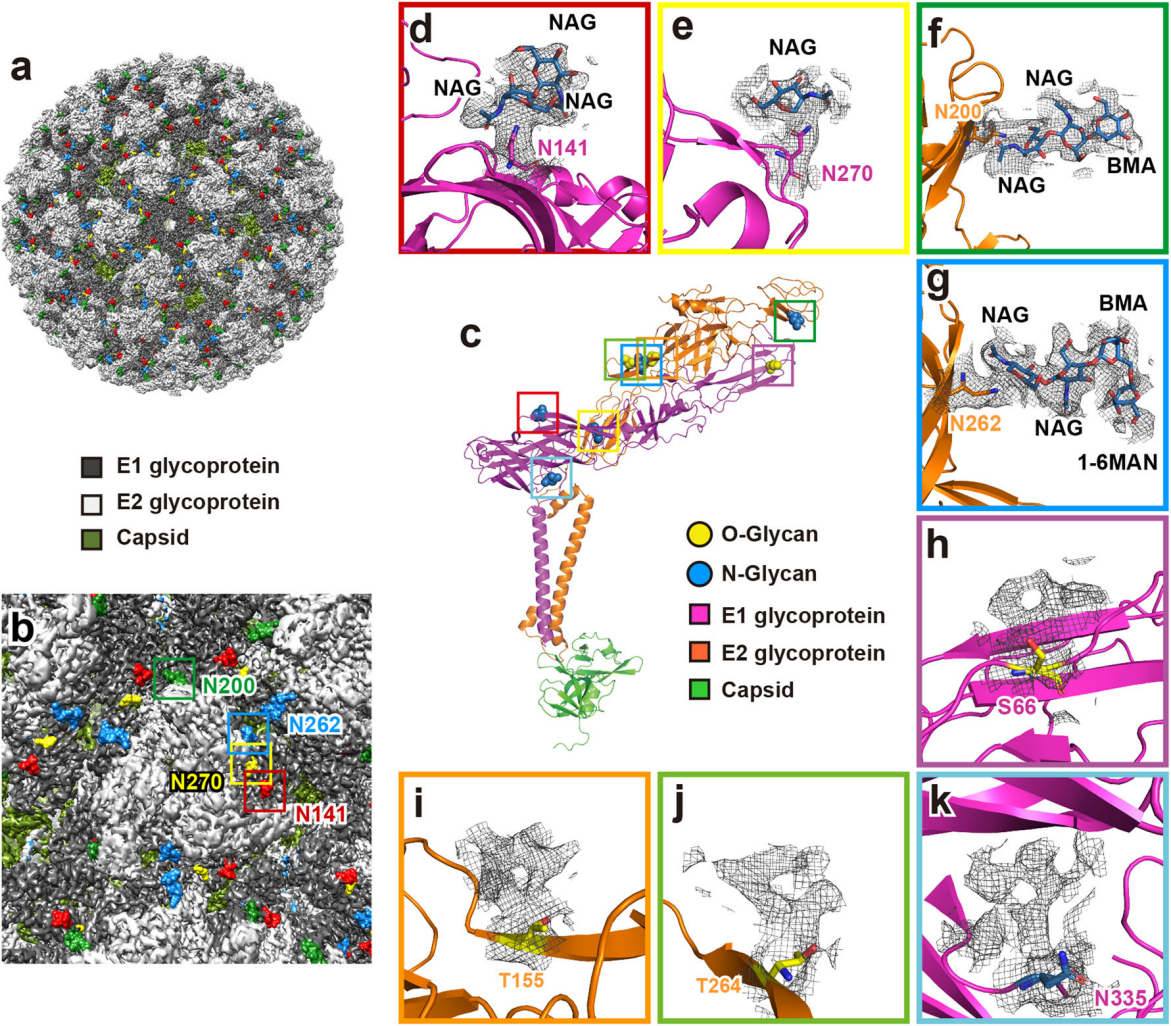
Atomic models of glycans fitted in the density maps in the E1–E2 ectodomain. **a** External surface of a GETV virion, with glycosylation sites displayed in color (E1 N141 in red, E1 N270 in yellow, E2 N200 in green, and E2 N262 in blue, respectively). **b** Zoomed-in view of the external surface close to the icosahedral 3-fold vertices; the glycosylation sites are framed and labeled using the same color scheme as shown in panel **a**. **c** Glycosylation sites in the atomic model of E1–E2–Capsid heterotrimer. **d**–**g** Zoomed-in views of *N*-glycosylation sites with residues shown as sticks (N141 and N270 in E1 are in purple, and N200 and N262 in E2 in orange). Cryo-EM densities attributed to glycans are shown as mesh; the atomic models for the glycans are shown as blue sticks. The names of carbohydrate monomers are labeled. *N*-Acetyl-glucosamine (NAG) and mannose (BMA: β-d-mannose; 1-6MAN: α-(1-6)-d-mannose) were assigned as the hexosamine and hexose monomers based on cryo-EM density map interpretation and models previously built for MAYV^25^. **h**–**k** Zoomed-in views of glycosylation sites with residues shown as sticks; cryo-EM densities attributed to glycans are shown in the mesh. Although protruding densities adjacent to S66, T155, T264, and N335 are strong, these did not support precise fitting for glycan atomic models.

Viral envelope proteins have evolved to be extensively glycosylated with versatile functions ranging from immune evasion by glycan shielding to enhancement of cell infection^36^. Glycosylation sites have been reported in CHIKV^34^, EEEV^24^, SINV^22^, and MAYV^25^. We first predicted the *N*-glycosylation and *O*-glycosylation sites on GETV E1/E2 using NetNGlyc^37^, NetOGlyc^38^, GlycoMine^39^, and YinOYang^37^. A list of the predicted glycosylation sites is provided in Supplementary Table S4. We next utilized a mass spectrometry method to identify glycosylation sites in the GETV E1 and E2 glycoproteins (Supplementary Figs. S17 and S18), and a list of the detected sites is given in Supplementary Table S4. In the 2.8 Å resolution cryo-EM density map, we sought the densities next to the residues which were not assigned to proteins. A total of eight densities were observed: four in E1, including S66, N141, N270, and N335. Another four in E2, T155, N200, T264, and N262 (Fig. 5 and Supplementary Fig. S19). Four densities for glycans were modeled and fitted associated with N141 and N270 of E1, and N200 and N262 of E2 (Fig. 5d–g and Supplementary Fig. S19a–d). Although protruding densities adjacent to S66, T155, T264, and N335 were strong, these did not support precise fitting for glycan atomic models (Fig. 5h–k and Supplementary Fig. S19e–h).

The N-glycosylation of E1 N141 and E2 N262 have also been reported in the MAYV structure, for which glycan models were built^25^. In our GETV structure, densities associated with E1 N141 and E2 N262 were both clearly visible, allowing the fitting of atomic models for multiple hexosamine and hexose monomers. The proximal portion of the density map associated with E2 N262 accommodated three carbohydrates, which were modeled as classical N-glycans NAG–NAG–BMA (NAG: *N*-Acetyl-glucosamine and BMA: β-D-mannose, Fig. 5g). At the position of the third carbohydrate BMA, one branch density was observed, in which a 1-6MAN (α-(1-6)-D-mannose) monomer was modeled and fitted (Fig. 5g). A similar model of NAG–NAG was fitted in the density proximal to E1 N141 (Fig. 5d). E2 N200 has a density that also fitted the typical *N*-glycans, NAG–NAG–BMA (Fig. 5f). The E1 N270 has a less defined density compared to E1 N141, E2 N200 or N262 where only one NAG monomer could be modeled (Fig. 5e). All four glycan moieties were surface-exposed (Fig. 5a, b), and they were accessible on the GETV surface close to the trimer/trimer interface (Fig. 5b). This surface exposure of glycosylation sites suggests their potential roles in evading detection by the host immune system, and/or in promoting attachment to the host cells to enhance viral uptake.

### S-acylation sites in the E1 and E2 TM helixes

S-acylation is a post-translational modification that attaches fatty acids, often palmitic acid, to cysteine residues; this modification occurs on both peripheral and integral membrane proteins^40^. These fatty acids increase a protein’s hydrophobicity and also facilitate interactions with lipid bilayers, and S-acylation is known to function directly in modulating proteins’ stability^40^. Protein acylation was originally discovered in the vesicular stomatitis virus^41^. Over the past 40 years, numerous viruses have been reported to undergo S-acylation, such as human immunodeficiency virus (HIV), SARS-CoV, influenza A virus, and hepatitis C virus (HCV)^42^. For alphaviruses, both SFV and SINV have been reported to contain S-acylation sites^43–46^. Using radioactivity derived from [^3^H]palmitic acid and [^3^H]stearic acid labeling, Veit et al., reported that SFV E1 contains mainly stearic acid, while E2 is acylated primarily with palmitic acid^43^. In a later study, Kordyukova et al. reported that SFV E1 is stoichiometrically acylated with stearic acid, whereas E2 contains palmitic acid and stearic acid at a ratio of 3:1, indicating that stearic acid was attached to only one cysteine^47^. In the 6 Å resolution cryo-EM structure of BFV, density was found next to residue C394 in the E2 TM helix; however, no atomic model containing a fatty acid was constructed^19^.

**Fig. 6:**
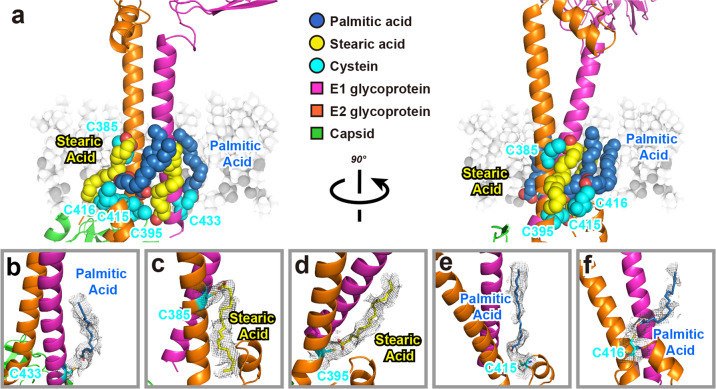
S-acylation sites were identified in the E1 and E2 TM helixes and in the E2 cytoplasmic tail. **a** Overview of S-acylation sites of E1 and E2 proteins. E1 and E2 are presented as cartoons (in Pymol) and are colored using the same color scheme as in Fig. 2. The involved cysteines, palmitic acids, and stearic acids are presented as spheres, colored with cyan, steal blue, and yellow, respectively. The inner leaflet of the viral envelope membrane is shown in the background. **b**–**f** Zoomed-in views of S-acylation sites. Palmitic acids and stearic acids are shown as sticks. Cryo-EM densities attributed to the cysteines, as well as palmitic acids and stearic acids, are shown as mesh.

In the present study, we first predicted the possible S-acylation sites using the predictors CSS-Palm and GPS-Palm^48,49^. A list of predicted S-acylation sites is given in Supplementary Table S5. Next, we utilized liquid chromatography–mass spectrometry to identify S-acylation sites in the GETV E1 and E2 proteins (Supplementary Figs. S20–S22), and a list of the sites is stated in Supplementary Table S5. Finally, in the 2.8 Å resolution GETV map, we examined the densities next to the residues. A total of five such densities were identified: one at E1 C433, and four in E2 (C385, C395, C415, and C416) (Fig. 6). According to the mass spectrometry analysis, stearic acids were attached to C385 and C395 in E2, while the other three cysteines, C433 in E1, C415 and C416 in E2, were acylated with palmitic acids. Therefore, we accommodated the atomic models of stearic acid in the straight proximal portion of the density next to E2 C385 and C395 (Fig. 6c, d), and fitted atomic models of palmitic acid in the densities adjacent to E1 C433 and E2 C415 and C416 (Fig. 6b, e, f). Compare the primary sequence between GETV and SFV, although the five cysteines were identical, the sequences around E1 C433 (Supplementary Fig. S14) and around E2 C395 were not fully conserved (Supplementary Fig. S15), suggesting that there could be a species diversification of S-acylation between GETV and SFV. As illustrated in Fig. 6a and Supplementary Fig. S23, the five highly or absolutely conserved C-terminal S-acylation sites strongly anchored the E1 and E2 TM helixes into the inner leaflet of the lipid bilayer of the viral envelope membrane. Considering the large portion of both the E1 and E2 ectodomain (Fig. 1h), it is likely that S-acylation in the TM helixes stabilizes the E1/E2 glycoproteins.

### Cholesterol molecules and a phospholipid molecule in the hydrophobic pocket increase the stability of the E1–E2 heterodimer

Previous studies have demonstrated that a hydrophobic pocket is essential for alphavirus assembly at the outer leaflet of the envelope membrane^22,25^. The hydrophobic pocket comprises the TM helix from E1, and the TM helix and domain D of E2 (Fig. 1h). In the 3.5-Å-resolution SINV structure, a “pocket factor” was identified; this was predicted to be a hydrophobic phospholipid tail^22^. In the 4.4-Å-resolution MAYV structure, a long density was identified in the hydrophobic pocket, and a C18 hydrocarbon lipid molecule, octadecane, was fitted into the density^25^. Moreover, pocket factors have been characterized in other viruses, such as Zika virus^50^ and Dengue virus^51^.

**Fig. 7:**
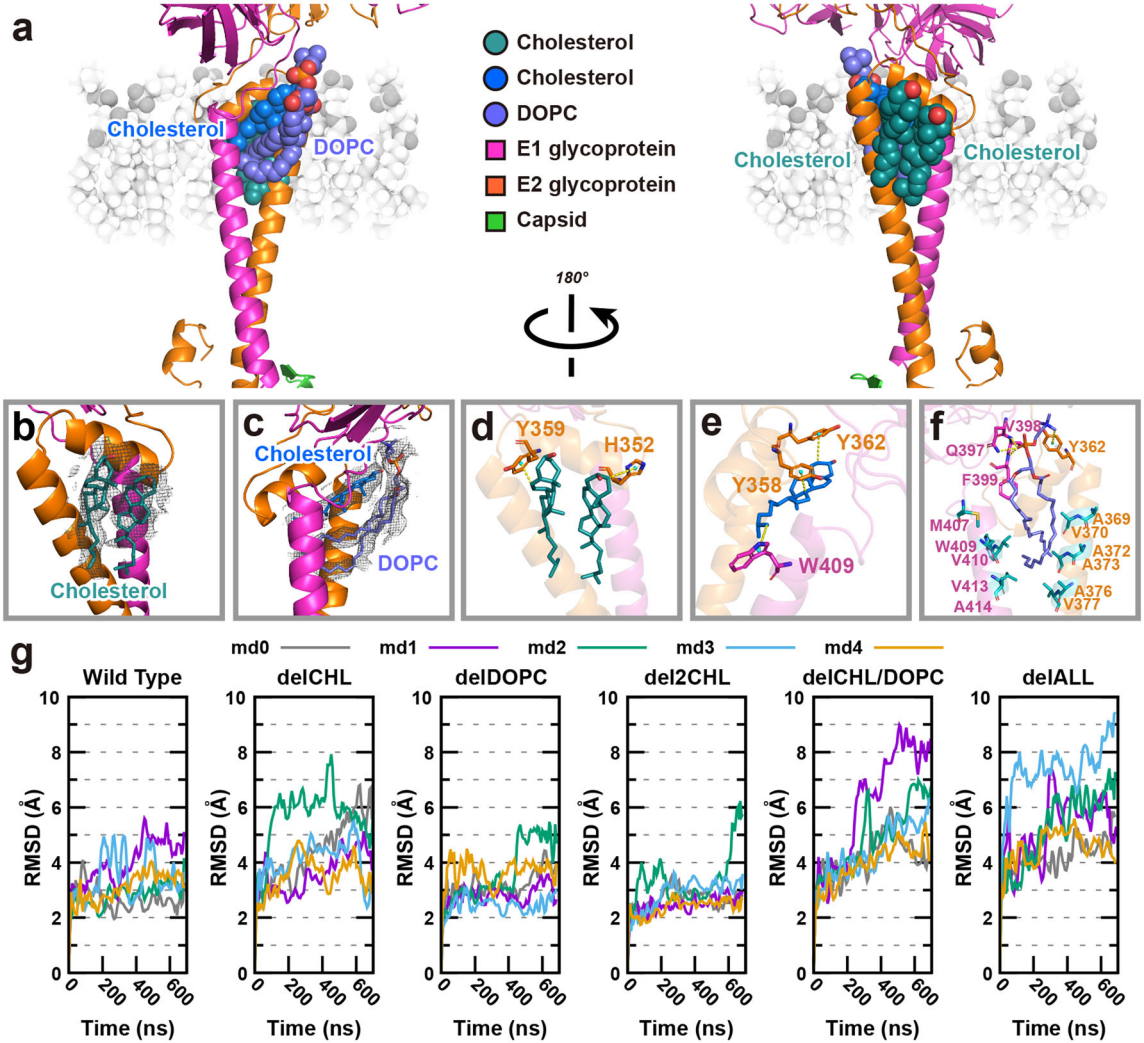
Cholesterol molecules and a phospholipid DOPC in the hydrophobic pocket. **a** Overview of the distribution of cholesterols and a phospholipid molecule DOPC. Cholesterol (blue) and DOPC (slate) are in the hydrophobic pocket formed by the E1 TM helix, the E2 TM helix, and E2 domain D. The other two cholesterols (deep teal) are positioned on the other side of domain D. E1 and E2 are presented as cartoons. The involved cholesterols and DOPC are presented as spheres, and the outer leaflet of the viral envelope membrane is shown in the background. **b**, **c** Zoomed-in views of cholesterols and DOPC molecules, which are shown as sticks. Cryo-EM densities attributed to the cholesterols and phospholipid are shown as mesh. **d**–**f** Zoomed-in views of the interactions between cholesterol or DOPC and E1 or E2. The aromatic residues positioned near the cholesterol or DOPC are shown as sticks with the same color as E1 or E2. The hydrophobic residues positioned near the tail of DOPC are shown as sticks. The center of mass of aromatic ring is shown as nb_spheres, colored in cyan. The yellow dashed lines represent the hydrogen bonds. A portion of the hydrogen bonds is CH–π hydrogen bonds between the soft acids CH and a soft base π-electron system. The remaining portion is common hydrogen bonds between the phosphate group of DOPC and Q397, V398 or F399. **g** Time evolutions from MD simulation analysis for the Cα-RMSD of E1 (295–435) and E2 (269–422) for systems “Wild Type”, “delCHL”, “delDOPC”, “del2CHL”, “delCHL/DOPC”, and “delALL”. All curves were smoothed with the Bezier method, implemented in gnuplot.

In our GETV structure, extra densities were clearly visible in the cavity formed by the E1 and E2 TM helixes, as well as in the region immediately adjacent to the helixes (Fig. 7a–c). Two cholesterols were modeled and fitted into the densities adjacent to the helixes (Fig. 7b), while one cholesterol was fitted in one density in the cavity (Fig. 7c). Cholesterol is the most abundant sterol in mammals, it is a major constituent of the plasma membrane and plasma lipoproteins. A previous study revealed that nearly 30% of the lipid class composition of baby hamster kidney cells (BHK, one of the most commonly used cell lines for expression of alphaviruses, and for GETV in the present study) represents cholesterol, and more than 40% of the envelope membrane of SFV particles produced in BHK cells are cholesterol^52^. Another density in the cavity displayed a “Y” shape, and a phospholipid molecule was modeled as “DOPC” (dioleoyl-phosphatidylcholine, Fig. 7c), with a hydrophilic “head” containing a phosphatidylcholine, and two hydrophobic “tails” derived from oleic acid, a mono-unsaturated omega-9 fatty acid. Both densities that were fitted with two oleic acid hydrocarbon tails had a kink that can accommodate the mono-unsaturated bond (Fig. 7c).

We subsequently analyzed the interactions between E1/E2 and cholesterol/DOPC in the hydrophobic pocket. Cholesterol molecules contain four fused carbon rings referred to as A, B, C, and D rings. The principal cholesterol–protein interactions occur through CH–π hydrogen bonds. CH–π bonds involve a soft acid CH and a soft base π-electron system^53^. As shown in Fig. 7d, in the cholesterol at the left, the B ring interacts with the aromatic ring of Tyr-359 in subdomain D, with mean distances of 4.0 Å. The A ring in the right cholesterol contacted with the aromatic ring of His-352, with a distance of 4.4 Å. This is consistent with the establishment of the cut-off values (4.5 Å) for the distance of the donor carbon atoms to the geometric center of the π–acceptor system^53^. Figure 7e displayed the cholesterol in the hydrophobic cavity, and the A and D rings interacted with Tyr-362 and Tyr-358, with mean distances of 4.3 Å and 3.8 Å, respectively. A CH–π hydrogen bond between a methyl group of DOPC and the aromatic ring of Tyr-362 were identified, with a distance of 4.5 Å. Moreover, hydrogen bonds between the phosphate group and Gln-397/Val-398/Phe-399 were confirmed. Beyond that, multiple hydrophobic residues were localized around the hydrophobic tails of DOPC. All of these interactions fixed DOPC in the hydrophobic pocket (Fig. 7f).

To further investigate how the cholesterol and DOPC molecules regulate the E1–E2 complex, we calculated the root mean square deviation of the Cα atom (Cα-RMSD) of E1–E2 against time in the six simulated systems using molecular dynamics (MD) simulations (Supplementary Videos S2–S7). As shown in Fig. 7g, the RMSD evolves differently in the six systems, with the overall values from the “delCHL/DOPC” (both cholesterol and DOPC in the pocket were removed) and the “delALL” (all three cholesterols and DOPC were removed) being the most unstable. The RMSD values increased drastically higher in these two systems with the magnitude reaching as high as 9 Å, whereas those in the “delCHL” (only cholesterol in the pocket was removed) system, the “delDOPC (only DOPC in the pocket was removed) system, and the “del2CHL” (two cholesterols surrounding the pocket were removed) system being similar to or only slightly higher than that in the Wild Type system. This indicates that the structure of the E1–E2 complex changes significantly whenever the pocket cholesterol and DOPC ligands were removed together, suggesting a cooperative role between the two pocket ligands in stabilizing the E1–E2 complex. Additionally, we calculated the number of native contacts within the E1–E2 complex during the evolution. As shown in Supplementary Fig. S24, the numbers of native contacts of the “delCHL/DOPC” and “delALL” systems overall decreased to under 4.9 × 10^4^, which was lower than those of the Wild Type system that decreased to ~5.0 × 10^4^. The systems “delCHL”, “delDOPC”, and “del2CHL” were similar to or slightly lower than those of the Wild Type system. These results show that the E1–E2 complex in the systems “delCHL/DOPC” and “delALL” lost more native contacts during simulation compared to other systems, indicating a larger structural change when the pocket cholesterol and DOPC ligands were removed together. The findings were quite similar to those presented in the Cα-RMSD analysis, which together suggests a cooperative role between the two pocket ligands in stabilizing the E1–E2 complex.

## Discussion

Viral glycosylation has broad roles in viral pathobiology, including mediating protein folding and trafficking, facilitating viral budding and release, directing immune evasion by glycan shielding, and shaping viral infection and tropism^36^. For alphaviruses, glycosylation is known to be a key determinant of cellular tropism and virulence: a lack of glycosylation in Ross River virus (RRV) renders virions unable to induce alpha/beta interferon production in myeloid dendritic cells, the professional antigen-presenting cells that rapidly respond to viruses, suggesting that viral glycans may negatively regulate antiviral responses^54^. Glycosylation has also been demonstrated to function in alphavirus infection and replication: a lack of glycosylation of the E1 protein in SINV severely impairs viral replication^55^. Further, a study showed that mutations of the glycosylation sites in the Salmonid alphavirus E1 and E2 proteins severely altered the virulence and production of an infectious virus^56^.

Previous structural analyses have revealed glycosylation sites in alphaviruses. A crystal structure of the mature E3–E2–E1 glycoprotein complexes from CHIKV revealed three N-linked glycans: N12 in E3, N263 in E2, and N141 in E1^34^. The cryo-EM structure of MAYV identified two glycosylation sites: N141 in E1 and N262 in E2, with at least five carbohydrates modeled as NAG–NAG–BMA–MAN–MAN at the E2 N262 site and with the canonical NAG–NAG–BMA glycan sequence modeled at the E1 N141 site^25^. Both the E1 N141 and E2 N262 sites are conserved in CHIKV, MAYV, SFV, RRV and GETV. Glycosylation sites were also identified in SINV by cryo-EM, including N139 and N245 in E1, N283 in E2, and N14 in E3, all of which displayed extra density next to an asparagine residue, although no glycan models were built^22^. In the present study, six new glycosylated residues — E1 S66, N270, and N335 along with E2 T155, T264, and N200 — were identified, and we built two more atomic models of the glycans (N270 and N200) precisely into the 2.8 Å cryo-EM density map (Fig. 5).

Protein S-acylation is a post-translational lipid modification wherein fatty acids, usually palmitic acid, are attached to cysteine (S-palmitoylation). There are also known examples of modifications of serine and threonine residues (O-palmitoylation)^42^. S-acylated proteins are typically membrane proteins, S-acylation enhances protein hydrophobicity and impacts protein subcellular localization, trafficking, stability, and interactions with other proteins^57^. S-acylation of viral proteins is known to be involved in virus assembly and infection. For example, the Spike protein of SARS-CoV, and quite recently, of SARS-CoV-2, have been shown to be palmitoylated; and these modifications have been associated with the cell–cell fusion process known to be essential for viral infectivity^57,58^. For alphaviruses, both E1 and E2 glycoproteins of SFV have been reported to contain S-acylation sites: E1 contains mainly stearic acid, while E2 is acylated primarily with palmitic acid^43^. Palmitoylation of cysteine residue in the E2 protein of SFV was demonstrated as an essential determinant for viral budding^44^. The E1 and E2 protein of SINV also have palmitoylation sites, and site-directed mutagenesis of cysteines in E1 and E2 abolished fatty acid attachment, and result in aberrant viral assembly and particle formation, slow replication at early times postinfection, and increased sensitivity to detergents^45,46^. Those findings supported an earlier study, which indicated that mutations in the SINV E2 glycoprotein led to defects in palmitic acid attachment, as well as defects in virus assembly and budding^59^. In the cryo-EM structure determination of BFV at 6 Å resolution, density was detected next to one cysteine residue in the E2 protein (C394); however, no atomic model containing a fatty acid was constructed^19^. In another cryo-EM study of VEEV at 4.4 Å resolution, three conserved E2 cysteine residues (C396, C416, and C417) were mapped near the lipid head groups of the inner membrane; note that no density attributable to S-acylated fatty acids was found^20^.

Cholesterol is an essential structural component of cell membranes and can also serve as a structural component of viral envelope membranes. The membrane of mammalian-derived SFV virions comprises more than 40% cholesterol^52^. Interestingly, the cholesterol content in the envelope of MAYV particles obtained from mammalian cells is 10-fold higher in cholesterol content as compared to viruses produced in mosquito cells^60^. A similar discrepancy in cholesterol content was found in SINV grown in mammalian vs in insect cells^61^. In all studies, BHK cells were used as the mammalian host cells; these are the same cells we used in the present study to propagate and purify GETV. Thus, it was not surprising that cholesterol was present in the GETV envelope membrane. It was however surprising that we detected one cholesterol molecule in the hydrophobic pocket and two cholesterol molecules surrounding the TM helixes of the E1 and E2 proteins, which indicated that cholesterol directly interacts with the E1 and E2 proteins (Fig. 7 and Supplementary Fig. S25). Several studies have demonstrated the impacts of cholesterol binding to viral proteins: cholesterol directly interacts with the cytoplasmic tail of the HIV glycoprotein gp41, and cholesterol is necessary for driving lateral self-aggregation and clustering of the envelope glycoproteins, therefore facilitating HIV fusion with the host cell and consequent viral entry^62^. A solid-state NMR study has revealed that two cholesterol molecules bind to the influenza M2 protein. Furthermore, cholesterol-mediated M2 clustering to the budding virus induced the necessary curvature for membrane scission^63^. Cholesterols also interact with the glycoprotein hemagglutinin of the influenza virus, which is essential for virus replication and affects virus assembly^64^.

Several studies have also addressed the potential impacts of cholesterol present in viral envelope membranes, depletion of cholesterol from envelopes has been shown to abolish the infectivity of influenza virus, human herpesvirus, herpes simplex virus, hepatitis C virus, and HIV^65–68^. For the alphaviruses CHIKV, SFV, SINV, and MAYV, cholesterol is required for viral entry to host cells^69–72^. After entry, viral RNA is released and replicated; this process also depends on intracellular membranes, and cholesterol impacts on viral replication: SINV replication was faster and reached higher titer in cholesterol-rich fibroblasts, and virions produced in cholesterol-rich fibroblasts are more infectious than viral particles produced from normal human fibroblasts^73^. Similarly, drugs that induced intracellular cholesterol accumulation and affected cholesterol biosynthesis, conferred strong inhibition against CHIKV replication^74^. Notably, gene expression profiling of cells infected with the M1 strain of GETV (which was isolated from *Culex* mosquitoes and is known to infect horses and pigs^75^), demonstrated that more than 60% of genes associated with the cholesterol biosynthesis pathway are downregulated after GETV M1 infection^76^. Taken together, cholesterol is not only a molecular factor that essential for alphavirus entry, replication, budding, and exit from host cells, but also a structural factor contributing to the stability of viral hydrophobic pocket in the envelope membrane.

## Materials and methods

### Virus infection

All protocols were approved by the Institutional Animal Care and Use Committee of Shanghai Tenth People’s Hospital. Mice were healthy and had no serum neutralizing antibody against GETV before the experiment. Newborn mice (2-day old or 3-day old) were inoculated oronasally with 10 μL (10^6^ TCID_50_/mL, 50% tissue culture infectious dose) GETV-V1 strain. Pregnant mice were inoculated oronasally with 100 μL (10^6^ TCID_50_/mL) GETV-V1 strain in early-gestation (E6), middle-gestation (E10), and late-gestation (E14).

### Virus production and purification

Mature and infective GETV virions were handled in BSL2 facilities at Cryo-Electron Microscopy Center, Southern University of Science and Technology, and at College of Animal Veterinary Medicine, Henan Agricultural University. GETV-V1 strain (GenBank Sequence Accession: KY399029.1) was incubated in monolayers Baby Hamster Kidney Fibroblast Cells (BHK-21, ATCC CCL-10) at an approximate multiplicity of infection (MOI) of 0.1 for 30 min to allow viruses to bind and enter into the BHK cells. The supernatant was removed and replaced with a fresh culture medium (DMEM with 2% FBS and 1% PS). GETV was proliferated in 10 T175 culture flasks and harvested 36 h postinfection. The cell debris of the supernatant was removed at 10,000× *g* for 1 h (Sorvall LYNX 6000 Superspeed Centrifuge). The GETV particles were concentrated by centrifuging at 80,000× *g* for 1.5 h in Ultracentrifuge (Beckman Optima XPN-100) with a type 45 Ti rotor at 4 °C. After soaking in 30 mL phosphate-buffered saline (PBS, pH 7.47) for 4 h, the virus suspension was initially purified via a 20% (w/v) sucrose cushion at 80,000× *g* for 1.5 h in a type SW32 Ti rotor at 4 °C. The virus pellet was gently resuspended in 2 mL PBS buffer for 4 h and loaded onto a linear 20%–50% (w/v) sucrose density gradient at 100,000× *g* for 2 h in an SW41 Ti rotor at 4 °C. The light scattering band corresponding to the virus particles was collected and resuspended in PBS. The integrity and purity of the GETV particles were examined by SDS-PAGE and mass spectrometry, and by negative-stain electron microscopy.

### Cryo-EM sample preparation and data acquisition

A 5 μL virus sample was added to the glow-discharged 400-mesh grid covered with amorphous nickel-titanium alloy film (R2/1) at 100% humidity and 6 °C, blotted with filter paper and frozen by plunging into liquid ethane using a Vitrobot Mark IV system (Thermo Fisher Scientific Inc.). Cryo-EM data were collected on a 300 kV Titan Krios microscope (Thermo Fisher Scientific Inc.) equipped with a BioContinuum Imaging Filter (Gatan Inc.). The images were recorded on a K3 summit direct detection camera (Gatan Inc.) using SerilEM software for automated image acquisition. The images were recorded at a nominal magnification of 105,000× in super-resolution mode, yielding a calibrated pixel size of 0.83 Å. Each exposure was dose-fractionated into 32 frames leading to a total dose of 40 e^–^/Å^2^. The final defocus range of the micrographs was –0.8 to –1.5 μm.

### Image processing

A total of 16,894 movie stacks of the GETV were collected. The beam-induced drift was corrected using MotionCor2^77^. The contrast transfer function (CTF) parameters were estimated using CTFFIND4^78^. In total, 171,059 particles were extracted using the EMAN2^79^. The 4× binned particle images were subjected to reference-free 2D classification in RELION-3^80^. After several rounds of reference-free 2D classification, a subset of 144,254 particles was isolated for 3D classification and reconstruction. Two maps at 8.94 Å resolution and 9.54 Å resolution were obtained with 56,573 and 43,653 good particles, respectively. The coordinates were used to extract the 2× binned particles for 3D classification using RELION-3 with I4 symmetry imposed, which resulted in two maps at 6.64 Å resolution and 6.69 Å resolution obtained from 43,775 and 62,415 good particles, respectively. The rotational and translational parameters of each particle were further refined with jalign program from JSPR, which enabled the reconstruction of a better map at 4.1 Å resolution using the j3dr program in JSPR^81^. To overcome the defocus gradient and the heterogeneity of the ~70 nm virus, block-based reconstruction was performed to further improve the resolution^30^. Three types of blocks (five trimers near the icosahedral fivefold axis, four trimers near the icosahedral-three-fold axis and two trimers near the icosahedral twofold axis) were selected, 3D classified and refined separately. Maps at 3.05 Å, 3.28 Å, and 3.14 Å, were obtained using unbinned particles. At this stage, CTF refinement was carried out to better estimate the local defocus value for each block, a final round of 3D refinement generated density maps at a resolution of 2.81 Å, 2.92 Å, and 2.85 Å, respectively, as estimated by the gold-standard Fourier shell correlation (FSC) cut-off value of 0.143 (Supplementary Figs. S9 and S10). The local resolution distribution of the final reconstruction was assessed using ResMap^82^ (Supplementary Fig. S10).

### Model building and refinement

An asymmetric subunit of the VEEV model (PDB ID: 3J0C) was used as a template and rigid-body fitted into the 2.8 Å GETV map using UCSF Chimera^83^. The amino acid sequences of VEEV were then mutated to GETV and an initial 3D atomic model of E1–E2–Capsid was built using Coot^84^. The resulting GETV coordinates were then used as a starting point to a flexible refinement through the PHENIX^85^. The refinement cycle was repeated, and the quality of the final 3D atomic model of E1–E2–Capsid was evaluated using MolProbity^86^. The cryo-EM data collection and processing, block-based reconstruction, model building, and refinement statistics are summarized in Supplementary Table S1.

### Quantitative *N*-glycosylation analysis by LC-MS/MS

E1 and E2 gel bands were excised for LC-MS/MS for quantitative *N*-glycosylation analysis. Protein bands were dissolved by 200 μL of 8 M urea with 10 mM DTT and digested by 100 μL of 25 mM ammonium bicarbonate containing 0.01 μg/μL trypsin before being collected and lyophilized in a sterile centrifuge. The dried polypeptide was dissolved in 40 mM NH_4_HCO_3_ prepared with ^18^O. Two units of rPNGase F were defined as the amount of enzyme that digests 100 μg polypeptide. The lyophilized peptide fractions were resuspended in ddH_2_O containing 0.1% formic acid. The online Chromatography separation was performed on the EASY-nLC1200 system. The trapping and desalting procedure was carried out with 20 μL solvent A (0.1% formic acid). DDA mass spectrum techniques were used to acquire tandem MS data on a Thermo Fisher Q Exactiv Mass Spectrometer fitted with a Nano Flex ion source. For a full mass spectrometry survey scan, the target value was 3 × 10^6^ and the scan ranged from 350 to 2000 m/z at a resolution of 70,000. For the MS2 scan, only spectra with a charge state of 2–5 were selected for fragmentation by higher-energy collision dissociation. *N*-linked glycans can be released from the glycoprotein with the enzyme *N*-glycosidase F rPNGase F. Asparagine residues can cause a mass increase of 2.9882 Da in “heavy” water and identified the *N*-glycosylation sites by LC-MS/MS peptide mapping. The MS data were analyzed for protein modification using PEAKS Studio 8.5.

### Quantitative S-acylation analysis by LC-MS/MS

E1 and E2 gel bands were excised for LC-MS/MS for quantitative S-acylation analysis.100 μL of the decolorizing solution was added and placed at room temperature to decolor overnight. 100% acetonitrile was added until the gel mass turned white. 10 μL 0.01 μg/μL trypsin was added to the gel to fully absorb by icing until it became transparent. The peptides were analyzed using Easy-nLC nanoflow HPLC system connected to Orbitrap Fusion mass spectrometer (Thermo Fisher Scientific, San Jose, CA, USA). A total of 1 μg of each sample was loaded onto a Thermo Scientific EASY column (two columns) using an autosampler at a flow rate of 200 nL/min. The sequential separation of peptides on a Thermo Scientific EASY trap column (100 μm × 2 cm, 5 μm, 100 Å, C18) and analytical column (75 μm × 25 cm, 5 μm, 100 Å, C18) was accomplished using a segmented gradient from 5% to 28% Solvent B (0.1% formic acid in 100% ACN) for 40 min, followed by 28%–90% Solvent B for 2 min and then 90% Solvent B for 18 min. The column was re-equilibrated to its initial highly aqueous solvent composition before each round of analysis. The mass spectrometer was operated in positive ion mode, and MS spectra were acquired over a range of 375–1500 m/z. The resolving powers of the MS scan and MS/MS scan at 200 m/z for the Orbitrap Fusion were set as 120,000 and 60,000, respectively. The Data Dependent Mode was Top Speed, Cycle Time was 3 s and ions were fragmented through higher-energy collisional dissociation. The maximum ion injection times were set at 50 ms for the survey scan and 105 ms for the MS/MS scans, and the automatic gain control target values for the Master scan modes were set to 4e^5^ and for MS/MS was set to 1e^5^. The dynamic exclusion duration was 40 s. S-acylation modification occurs on CKST amino acids, for which the molecular weight change after modification is 238.23/266.26 Da (palmitic acid/stearic acid). The MS data were analyzed for protein modification using Proteome Discoverer 2.4.

### MD simulation study of cholesterols and DOPC regulation of E1–E2

The solved cryo-EM structure of the full-length E1–E2 complex was first truncated to keep only the amino acids of 396–438 for E1 and 340–403 for E2 as the starting protein model for MD simulations. The truncated protein (denoted as E1–E2), together with the ligands at three different compositions, was then packed with the lipid bilayer using the CHARMM-GUI tool^87^. The resulting systems simulated are E1–E2 with: (1) all the cholesterols and DOPC molecules retained (denoted as Wild Type); (2) removal of the pocket cholesterol only (denoted as delCHL); (3) removal of the pocket DOPC only (denoted as delDOPC); (4) removal of both cholesterol and DOPC in the pocket (denoted as delCHL/DOPC); (5) removal of the two cholesterols surround the pocket (denoted as del2CHL); and (6) all 3 cholesterols and DOPC molecule removed (denoted as delALL). The main lipid components with the same mixed lipid ratio in both inner and outer leaflets (DPPC/DOPC/POPC/DPPE/POPE/DPPS/POPS/Chol = 7.5:7.5:15:5:7:5:7:46) were used in constructing the membrane model following the reported lipidomic data^52^. The sphingomyelin was approximatively modeled by the POPC due to the lack of the force field parameters for sphingomyelin and the identical hydrophilic phosphorylcholine head group, similar conformation and charge distribution between the two molecules. In order to obtain a consistent protein–membrane packing conformation with the cryo-EM density data, the vector from the residue Val-410 of E1 to Lys-394 of E2 was aligned with the normal axis of the lipid bilayer when packing systems. Next, the constructed protein–ligand–membrane systems were submitted to the H ++ web server^88^ to determine the protonation states of the protein under the membrane environment in order to approximate a simulation pH of 7.0. As a result, the amino acids His-355 in E1 and His-352 in E2 were simulated as pronated. The protein was diagonally positioned in the *x–y* plane in the simulation box filled with the TIP3P water model^89^ to save space, leading to a box size of ~87 Å × 87 Å × 122 Å. The final systems were neutralized with the sodium ion and extra NaCl was added to model the experimental salt concentration of 0.15 M. All MD runs were performed using AMBER 19 package^90^ with the ff14SB protein force field^91^ and the LIPID17 lipid force field^92^. The van de Waals cutoff was set to 10 Å and PME was used for long-range electrostatic energy calculation. Minimization, heating and holding processes were carried out before the production run. All production simulations were performed at 310 K under the NPT ensemble with the integration step of 2 fs and an anisotropic pressure coupling. Five independent runs for each system of Wild Type, delCHL, delDOPC, delCHL/DOPC, del2CHL, or delALL were carried out to improve samplings, which led to a total of 30 simulations. Production trajectories of 700 ns were generated for all simulations. All figures and animations were prepared using gnuplot 5.4 and VMD^93^.

## Supplementary information

Supplementary Information

Video S1

Video S2

Video S3

Video S4

Video S5

Video S6

Video S7

## Data Availability

The atomic coordinates of E1–E2–Capsid of the GETV have been deposited in the Protein Data Bank under the accession code 7FD2. The cryo-EM maps of the envelope glycoprotein and capsid core, and the clipped virus at 5-fold, 3-fold and 2-fold have been deposited in the Electron Microscopy Data Bank under accession codes EMD-31533, EMD-31614, EMD-31615, and EMD-31617, respectively.
