# Supplementary material for: Structure of infective Getah virus at 2.8 Å resolution determined by cryo-electron microscopy
Source: Cell Discov. 2022 Feb 11;8:12. doi: 10.1038/s41421-022-00374-6 (PMC8832435; doi:10.1038/s41421-022-00374-6)
Supplement: Supplementary file 1 — Supplementary Information [file 41421_2022_374_MOESM1_ESM.pdf]

Supplementary Information

Supplementary Figure S1. GETV is a mosquito-borne arbovirus.

Supplementary Figure S2. GETV caused reproductive disorders in pregnant mice.

Supplementary Figure S3. GETV antigen was not detected in the brain or spinal cord in 7-day-old newborn and 2-month-old adult mice that were inoculated with GETV.

Supplementary Figure S4. Protein contents of purified GETV were analyzed by polyacrylamide gel electrophoresis.

Supplementary Figure S5. Mass spectrometry of GETV structural proteins.

Supplementary Figure S6. Mass spectrometry of GETV non-structural proteins.

Supplementary Figure S7. Negatively stained electron micrograph of GETV-V1 strain virions.

Supplementary Figure S8. Cryo-electron micrograph of purified GETV.

Supplementary Figure S9. Cryo-EM image-processing workflow.

Supplementary Figure S10. Local resolution of cryo-EM block-based reconstructions of GETV.

Supplementary Figure S11. Representative density and atomic models of each domain of E1-E2-capsid heterotrimer.

Supplementary Figure S12. Density maps of capsid.

Supplementary Figure S13. Interacting regions between GETV structural proteins.

Supplementary Figure S14. Multiple sequences alignment and secondary structural elements of alphaviruses E1 protein.

Supplementary Figure S15. Multiple sequences alignment and secondary structural elements of alphaviruses E2 protein.

Supplementary Figure S16. Multiple sequences alignment and secondary structural elements of alphaviruses capsid protein.

Supplementary Figure S17. Mass spectrometry of the N-glycosylation sites in GETV E1 protein.

Supplementary Figure S18. Mass spectrometry of the N-glycosylation sites in GETV E2 protein.

Supplementary Figure S19. Conserved glycosylation sites in the alphaviruses.

Supplementary Figure S20. Mass spectrometry of the S-acylation sites in the GETV E1 protein.

Supplementary Figure S21. Mass spectrometry of the S-acylation sites in the GETV E2 protein.

Supplementary Figure S22. MS spectra and table of mass shifts of S-acylation by LC-MS/MS.

Supplementary Figure S23. Transmembrane helices of E1 and E2.

Supplementary Figure S24. Time evolutions from MD simulation analyses for the native contacts within the E1-E2 complex.

Supplementary Figure S25. Comparison of the hydrophobic pocket between our GETV model and other alphavirus structures.

Supplementary Table S1. Cryo-EM data collection and processing, block-based reconstruction, model building, and refinement statistics.

Supplementary Table S2. Interaction areas between GETV structural proteins.

Supplementary Table S3. Protein-protein interactions in GETV and other alphaviruses.
Supplementary Table S4. Glycosylation sites in the E1 and E2 proteins.
Supplementary Table S5. S-acylation sites in the E1 and E2 proteins.

Supplementary Video S1. GETV caused mobility impairments in pelvic limbs in newborn mice.

Supplementary Video S2. Molecular dynamics simulation of interactions in the Wild Type E1-E2 system.

Supplementary Video S3. Molecular dynamics simulation of interactions in the "delCHL" (only cholesterol in the pocket was removed) system.

Supplementary Video S4. Molecular dynamics simulation of interactions in the "delDOPC (only DOPC in the pocket was removed) system.

Supplementary Video S5. Molecular dynamics simulation of interactions in the "del2CHL" (2 cholesterols surround the pocket were removed) system.

Supplementary Video S6. Molecular dynamics simulation of interactions in the "delCHL/DOPC" (both cholesterol and DOPC in the pocket were removed) system.

Supplementary Video S7. Molecular dynamics simulation of interactions in the "delALL" (all three cholesterols and DOPC were removed) system.

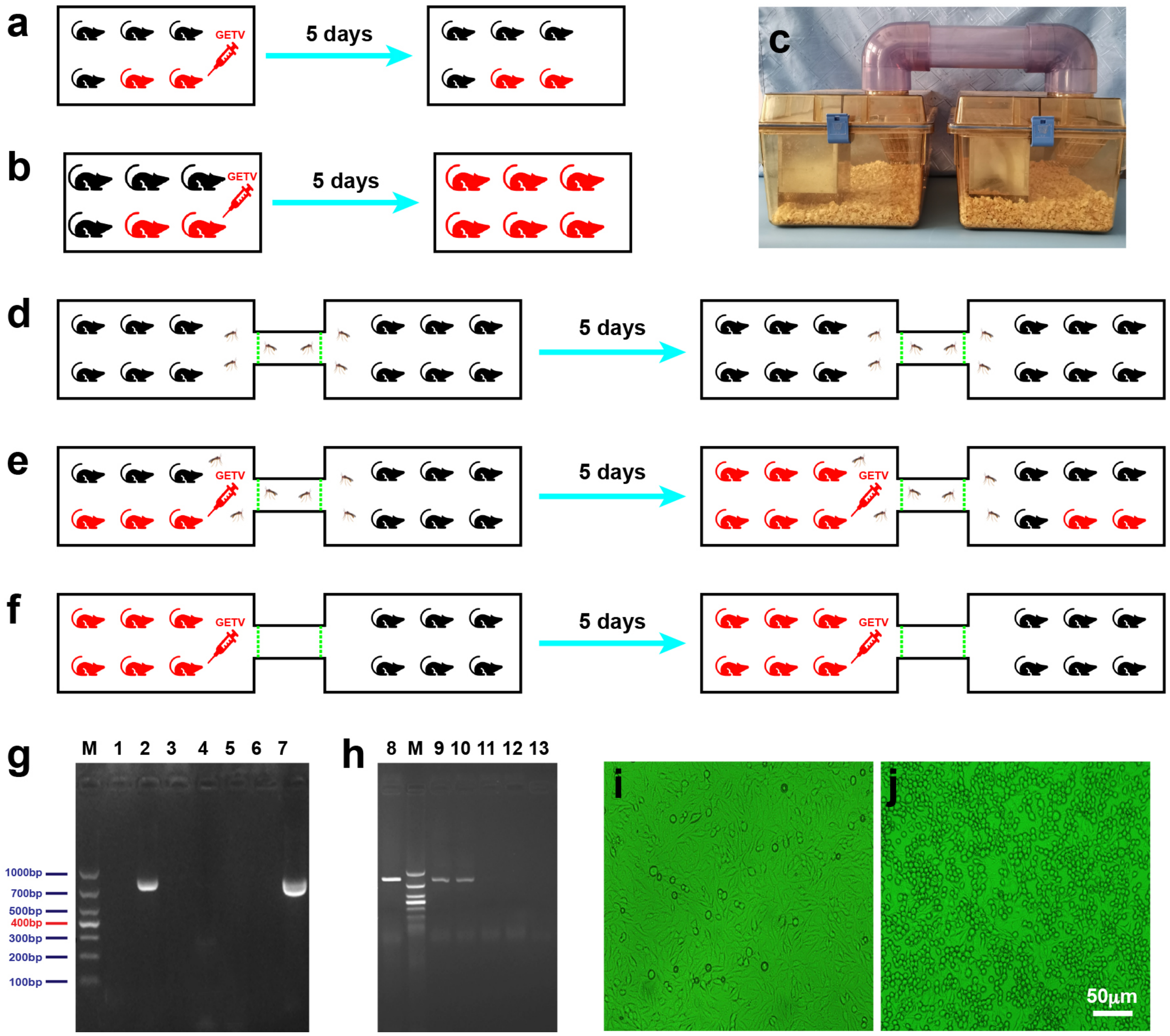

Supplementary Figure S1. GETV is a mosquito-borne arbovirus.

a, Photo of the device that was used in experiments **d** – **f**. **b** – **f**, Schematic illustrations of the experimental design to investigate the infectiousness of GETV. **b**, Experiments investigate the infectiousness of GETV between newborn mice. **c**, Experiments investigate the infectiousness of GETV between male adult mice. **d** – **f**, Experiments investigate GETV as a mosquito-borne arbovirus, not an airborne virus. **g** and **h**, Total RNA was extracted from tissue, including spleen, lung, cerebral cortex, and various lymph nodes using TRIzol reagent after 5 days post-inoculation (DPI)[1], and subjected to RT-PCR for GETV detection using specific primers (GETV-F: 5 ′ -

ACCGAAGAAGCCGAAGAA-3′, and GETV-R: 5′-GCACTCRAGGTCATACTTG-3′)[2]. M, markers; Lane 1, the buffer used in RT-PCR; Lane 2, supernatant of GETV cultured in BHK21 cells (positive control); Lane 3, test in mosquitos used in **d**; Lane 4 and 5, test in mice in the right-hand cage at 5 DPI in **d**; Lanes 6 and 7, GETV-free mice and infected mice in the cage at 5 DPI in **b**; Lane 8, GETV-free mice at 5 DPI in **c**, showing GETV positive, indicating the GETV could be transmitted via an animal bite. Lanes 9 and 10, GETV-free mice in the right-hand cage in **e**, showing positive and negative at 5 DPI, demonstrating GETV transmitted from mice in the left-hand cage via mosquitos. Lanes 11-13, three mice in the right-hand cage in **f** tested negative, revealed that GETV is not an airborne virus. Tissue samples from the GETV-free (**i**) and GETV-infected mice (**j**) in a right-hand cage in experiment **e** were cultured in BHK21 cells, images were captured after 35 hours. Cells infected with GETV (**j**) obviously developed morphological characteristics, such as enlarged, rounded, refractile, pyknosis, and detachment cells.

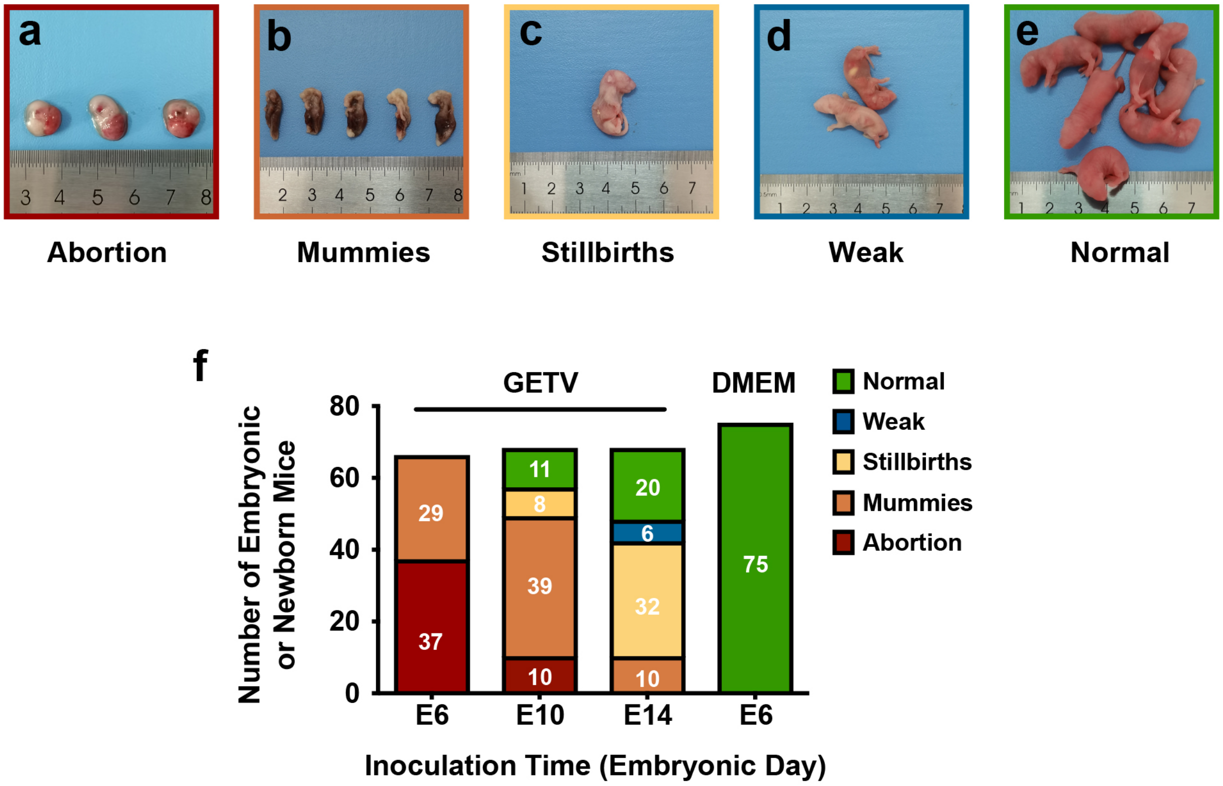

Supplementary Figure S2. GETV caused reproductive disorders in pregnant mice.

Twenty pregnant mice were randomly divided into four groups, and mice in the first three groups were inoculated oronasally with 100 μl (10^6 $TCID_{50}$/mL) GETV V1 strain at embryonic day 6 (E6, early-gestation), E10 (middle-gestation), and E14 (late-gestation), respectively. Mice in the fourth group were inoculated with an equal volume of DMEM at E6 as a control. Abortions (**a**, embryonic were expelled from the uterus before parturition), mummies (**b**, embryos were lost before farrowing), and stillbirths (**c**, embryos were lost around the time of birth, and might be prepartum or intrapartum) arising from GETV-infection were dominant in the E6, E10, and E14 groups. Some weak (**d**) and normal (**e**) newborns were found in E10 and E14 groups. **f**, Summary of the proportion of embryonic or newborn mice in the four groups. Numbers in the color frames in each column represented the number of mice that have each reproductive disorder.

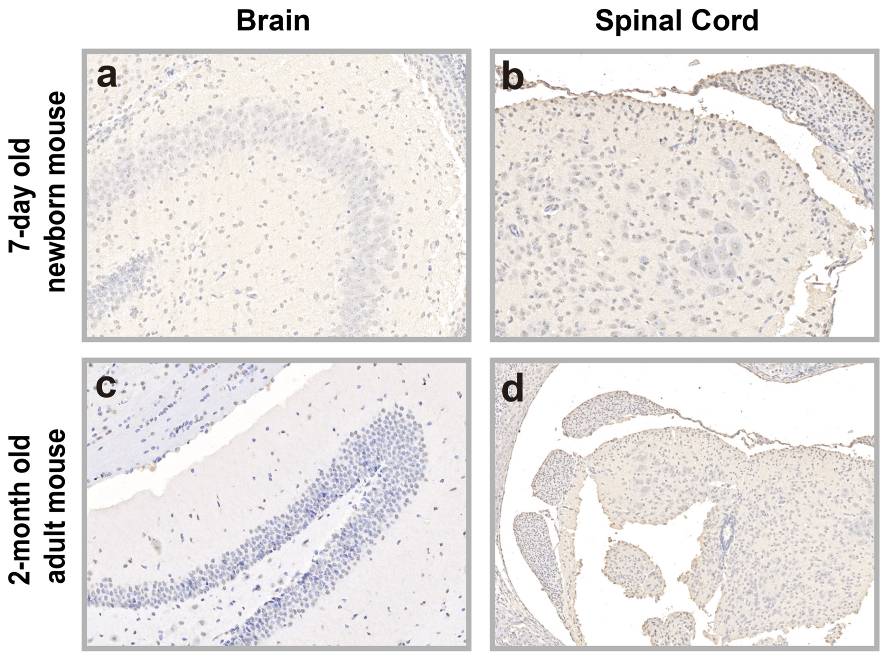

Supplementary Figure S3. GETV antigen was not detected in the brain or spinal cord in 7-day-old newborn and 2-month-old adult mice that were inoculated with GETV.

Forty 7-day-old newborn and forty 2-month-old adult mice were randomly divided into two groups, inoculated oronasally with 10 μl ($10^6 TCID_{50}$/mL) GETV strain GETV-V1 or control DEME. Mice were held under clinical observation after inoculation. **a** and **b**, No GETV antigen was detected in the hippocampal dentate gyrus of the brain or spinal cord from 7-day-old mice 3 DPI. **c** and **d**, No GETV antigen was found in 2-month-old mice 3 DPI. Monoclonal antibody against capsid protein of GETV was used for immuno-histochemical assays.

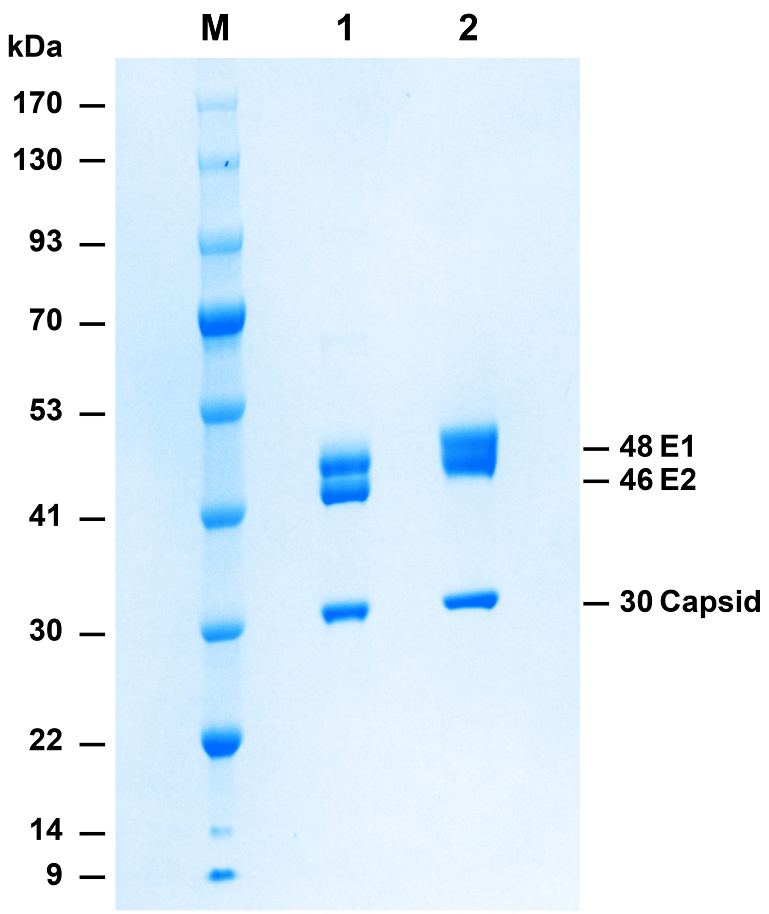

Supplementary Figure S4. Protein contents of purified GETV were analyzed by polyacrylamide gel electrophoresis.

Purified GETV samples were separated by 4-12% gradient SDS-PAGE (Bio-Rad) and the protein contents were stained with Coomassie brilliant blue. Three major structural proteins, E1, E2, and Capsid, are clearly identified. Lane M, protein markers; Lane 1, GETV proteins were separated under non-reducing conditions; Line 2, proteins were separated under reducing conditions.

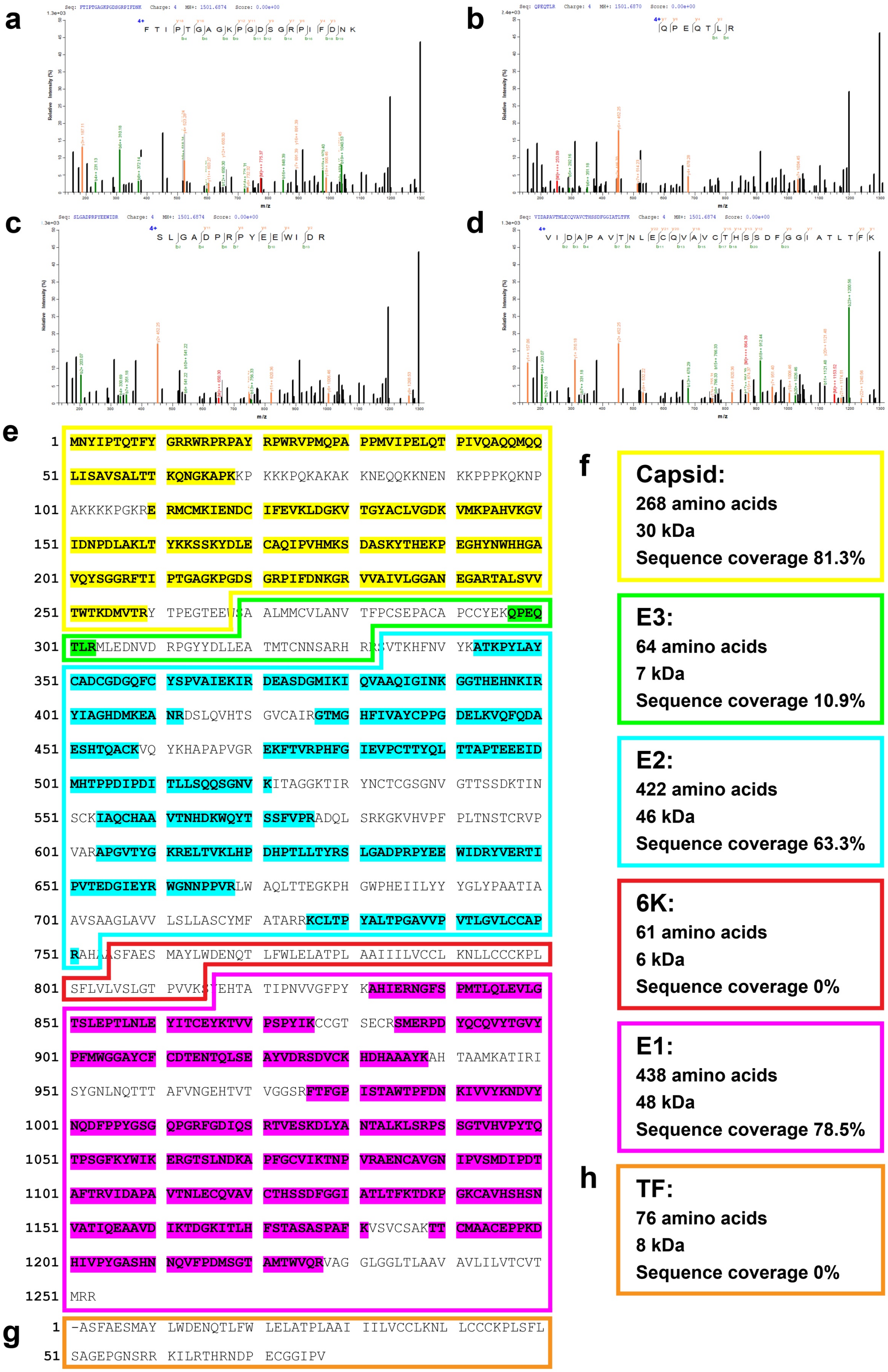

Supplementary Figure S5. Mass spectrometry of GETV structural proteins.

The protein contents of purified GETV samples were further analyzed with protein mass spectrometry. Representative peptide spectra from Capsid (**a**), E3 (**b**), E2 (**c**) and E1 (**d**). **e**, Four GETV-encoded structural proteins previously described were identified by mass spectrometry. **f,** Capsid, E1, and E2 that were highly abundant in SDS-PAGE analyses were displayed a higher score of sequence coverage, 81.3%, 78.5%, and 63.3%, respectively. Only one small peptide from the structural protein E3 was identified, and sequence coverage is 10.9%. Peptide from the structural protein 6K was not detected. **g**, The *6K* gene produced two distinct protein products, 6K and transframe (TF). This occurred via a (−1) ribosomal frameshift site that is highly conserved across the alphaviruses. The 6K and TF proteins each contain an identical N-terminal transmembrane domain (first 49 amino acids) and unique C-terminal ends[3]. **h**, Peptide from protein TF was not detected.

a

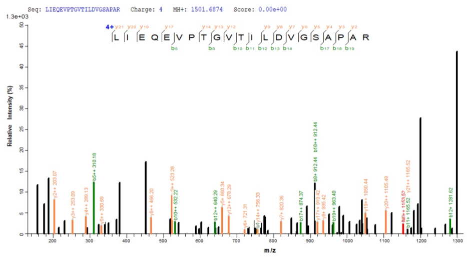

b

1	MKVTVDVEAD	SPFLKALQKA	FPAFEVESQQ	VTPNDHANAR	AFSHLATKLI
51	EQEVPTGVTI	LDVGSAPARR	LMSDHTYHCI	CPMKSAEDPE	RLANYARKLA
101	KASGTVLDKN	VSGKITDLQD	VMATPDLESP	TFCLHTDETC	RTRAEVAVYQ
151	DVYAVHAPTS	LYHQAIKGVR	TAYWIGFDTT	PFMFEALAGA	YPAYSTNWAD
201	EQVLQARNIG	LCATGLSEGR	RGKLSIMRKK	CLRPSDRVMF	SVGSTLYTES
251	RKLLRSWHLP	SVFHLKGKNS	FTCRCDTVVS	CEGYVVKKIT	ISPGIYGKTV
301	DYAVTHHAEG	FLMCKITDTV	RGERVSFPVC	TYVPATICDQ	MTGILATDVT
351	PEDAQKLLVG	LNQRIVVNGR	TQRNTNTMKN	YLLPVVAQAF	SKWAREARAD
401	MEDEKPLGTR	ERTLTCCCLW	AFKSHKIHTM	YKRPETQTIV	KVPSTFDSFV
451	IPSLWSSSLS	MGIRQRIKLL	LSARMAQGLP	YSGDRTEARA	AEEEEKEVQE
501	AELTRAALPP	LVSGSCADDI	AQVDVEELTF	RAGAGVVETP	RNALKVTPQA
551	HDHLIGSYLI	LSPQTVLKSE	KLAPIHPLAE	QVTVMTHSGR	SGRYPVDKYD
601	GRVLIPTGAA	IPVSEFQALS	ESATMVYNER	EFINRKLHHI	ALYGPALNTD
651	EESYEKVRAE	RAETEYVFDV	DKKACIKKEE	ASGLVLTGDL	INPPFHEFAY
701	EGLKIRPAAP	YHTTIIGVFG	VPGSGKSAII	KNMVTTRDLV	ASGKKENCQE
751	IMNDVKRQRG	LDVTARTVDS	ILLNGCKKGV	ENLYVDEAFA	CHSGTLLALI
801	ALVRPSGKVV	LCGDPKQCGF	FNLMQLKVHY	NHNICTRVLH	KSISRRCTLP
851	VTAIVSTLHY	QGKMRTTNRC	NTPIQIDTTG	SSKPASGDIV	LTCFRGWVKQ
901	LQIDYRGHEV	MTAAASQGLT	RKGVYAVRQK	VNENPLYSPL	SEHVNVLLTR
951	TENRLVWKTL	SGDPWIKVLT	NVPRGDFSAT	LEEWHEEHDG	IMRVLNERPA
1001	EVDPFQNKAK	VCWAKCLVQV	LETAGIRMTA	DEWNTILAFR	EDRAYSPEVA
1051	LNEICTRYYG	VDLDSGLFSA	QSVSLFYENN	HWDNRPGGRM	YGFNHEVARK
1101	YAARFPFLRG	NMNSGLQLNV	PERKLQPFSA	ECNIVPSNRR	LPHALVTSYQ
1151	QCRGERVEWL	LKKIPGHQML	LVSEYNLVIP	HKRVFWIAPP	RVSGADRTYD
1201	LDLGLPMDAG	RYDLVFVNIH	TEYRQHHYQQ	CVDHSMRLQM	LGGDSLHLLR
1251	PGGSLLMRAY	GYADRVSEMV	VTALARKFSA	FRVLRPACVT	SNTEVFLLFS
1301	NFDNGRRAVT	LHQANQKLSS	MYACNGLHTA	GCAPSYRVRR	ADISGHSEEA
1351	VVNAANAKGT	VSDGVCRAVA	KKWPSSFKGA	ATPVGTAKMI	RADGMTVIHA
1401	VGPNFSTVTE	AEGDRELAAA	YRAVASIIST	NNIKSVAVPL	LSTGTFSGGK
1451	DRVMQSLNHL	FTALDATDAD	VVIYCRDKNW	EKKIQEAIDR	RTAIELVSED
1501	VTLETDLVRV	HPDSCLVGRN	GYSATDGKLY	SYLEGTRFHQ	TAVDMAEIST
1551	LWPRLQDANE	QICLYALGET	MDSIRTKCPV	EDADSSTPPK	TVPCLCRYAM
1601	TAERVARLRM	NNTKNIIVCS	SFPLPKYRIE	GVQKVKCDRV	LIFDQTVPSL
1651	VSPRKYIQQP	PEQLDNVSLT	STTSTGSAWS	FPSETTYETM	EVVAEVHTEP
1701	PIPPPRRRRA	AVAQLRQDLE	VTEEIEPYVT	QQAEIMVMER	VATTDIRAIP
1751	VPARRAITMP	VPAPRVRKVA	TEPPLEPEAP	IPAPRKRRTT	STSPPHNPED
1801	FVPRVPVELP	WEPEDLDIQF	GDLEPRRRNT	RDRDVSTGIQ	FGDIDFNQSX
1851	LGRAGYIFS	SDTGPGHLQQ	KSVRQHELPC	ETLYAHEDER	IYPPAFDGEK
1901	EKVLQAKMQM	APTEANKSRY	QSRKVENMKA	LIVERLREGA	KLYLHEQTDK
1951	VPTYTSKYPR	PVYSPSVDDS	LSDPEVAVAA	CNSFLEENYP	TVANYQITDE
2001	YDAYLDLVDG	SESCLDRATF	CPAKLRCYPK	HHAYHQPQIR	SAVPSPFQNT
2051	LQNVLAAATK	RNCNVTQMRE	LPTMDSAVFN	VESFKKYACT	GEYWQEFKDN
2101	PIRITTENIT	TYVAKLKGPK	AAALFAKTHN	LVPLQEVPMD	RFVMDMKRDV
2151	KVTPGTKHTE	ERPKVQVIQA	AEPLATAYLC	GIHRELVRRL	KAVLTPNIHT
2201	LFDMSAEDFD	AIIAAHFQPG	DAVLETDIAS	FDKSQDDSLA	LTALMLLEDL
2251	GVDQELLDLI	EAAFGEITSV	HLPTGTRFKF	GAMMKSGMFL	TLFINTLLNI
2301	VIACRVLRDK	LSSSACAAFI	GDDNIVHGVR	SDPLMAERCA	SWVNMEVKII
2351	DATMCEKPPY	FCGGFILYDS	VAGTACRVAD	PLKRLFKLGK	PLPADDNQDE
2401	DRRRALKDET	VKWSRIGLRE	ELDVALSSRY	QVSGVGNITR	AMSTLSKSLK
2451	SFRKIRGPII	HLYGGPK			

c

nsP1:
534 amino acids
59 kDa
Sequence coverage 7.9%

nsP2:
798 amino acids
90 kDa
Sequence coverage 0%

nsP3:
523 amino acids
58 kDa
Sequence coverage 0%

nsP4:
612 amino acids
69 kDa
Sequence coverage 0%

Supplementary Figure S6. Mass spectrometry of GETV non-structural proteins.

a, Representative peptide spectrum of non-structural-protein 1 (nsP1). **b** and **c**, Only three peptides were identified in nsP1, and sequence coverage for nsP1 is 7.9%. Peptides from nsP2, nsP3 or nsP4 were not detected.

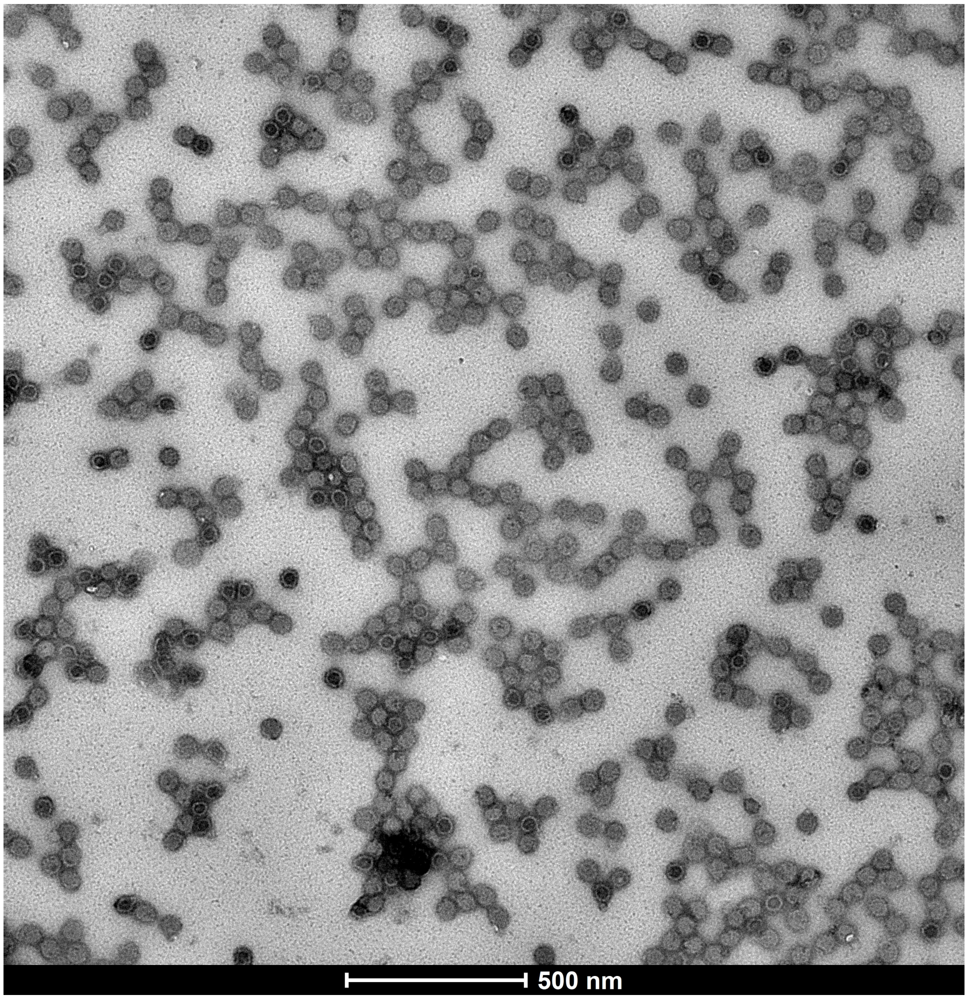

Supplementary Figure S7. Negatively stained electron micrograph of GETV-V1 strain virions.

Sucrose gradient purified GETV were negatively stained and imaged at 17,500X magnification.

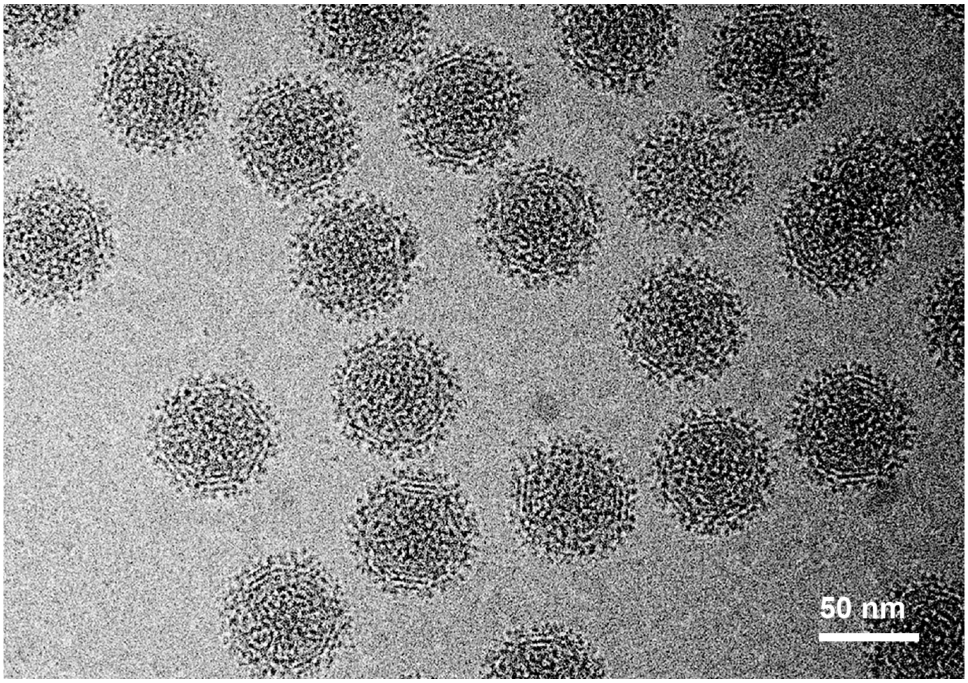

Supplementary Figure S8. Cryo-electron micrograph of purified GETV virions.

Micrographs were captured in a Thermo Fisher Scientific Titan Krios Cryo-Transmission Electron Microscope equipped with a Gatan K3 direct electron detector camera. Beam-induced shifting that blurred the captured images were corrected using MotionCor2[4].

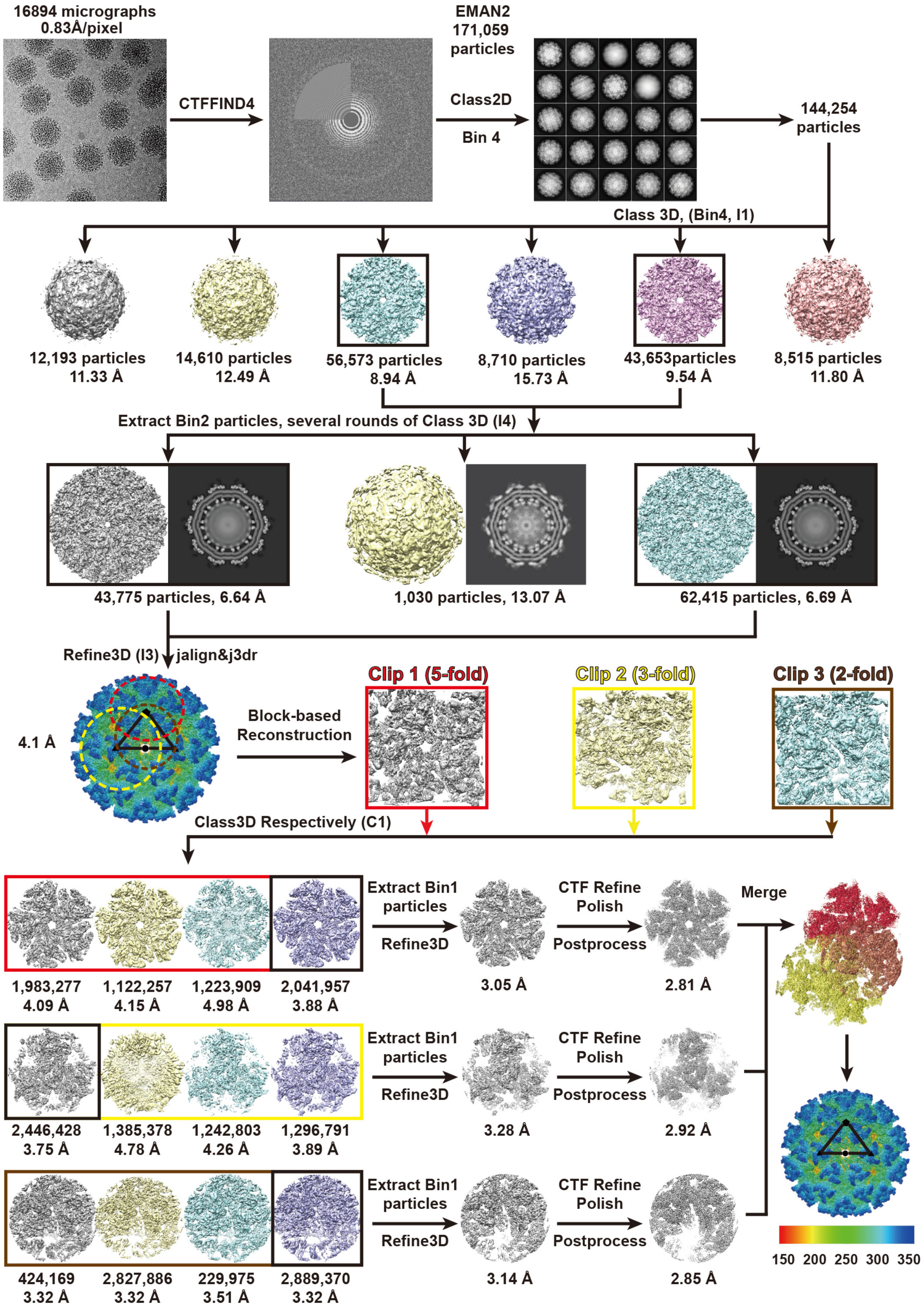

Supplementary Figure S9. Cryo-EM image-processing workflow.

Schematic of pre-processing, 2D and 3D classification, block-based reconstruction, and refinement procedures used to generate the 2.8-Å resolution density map.

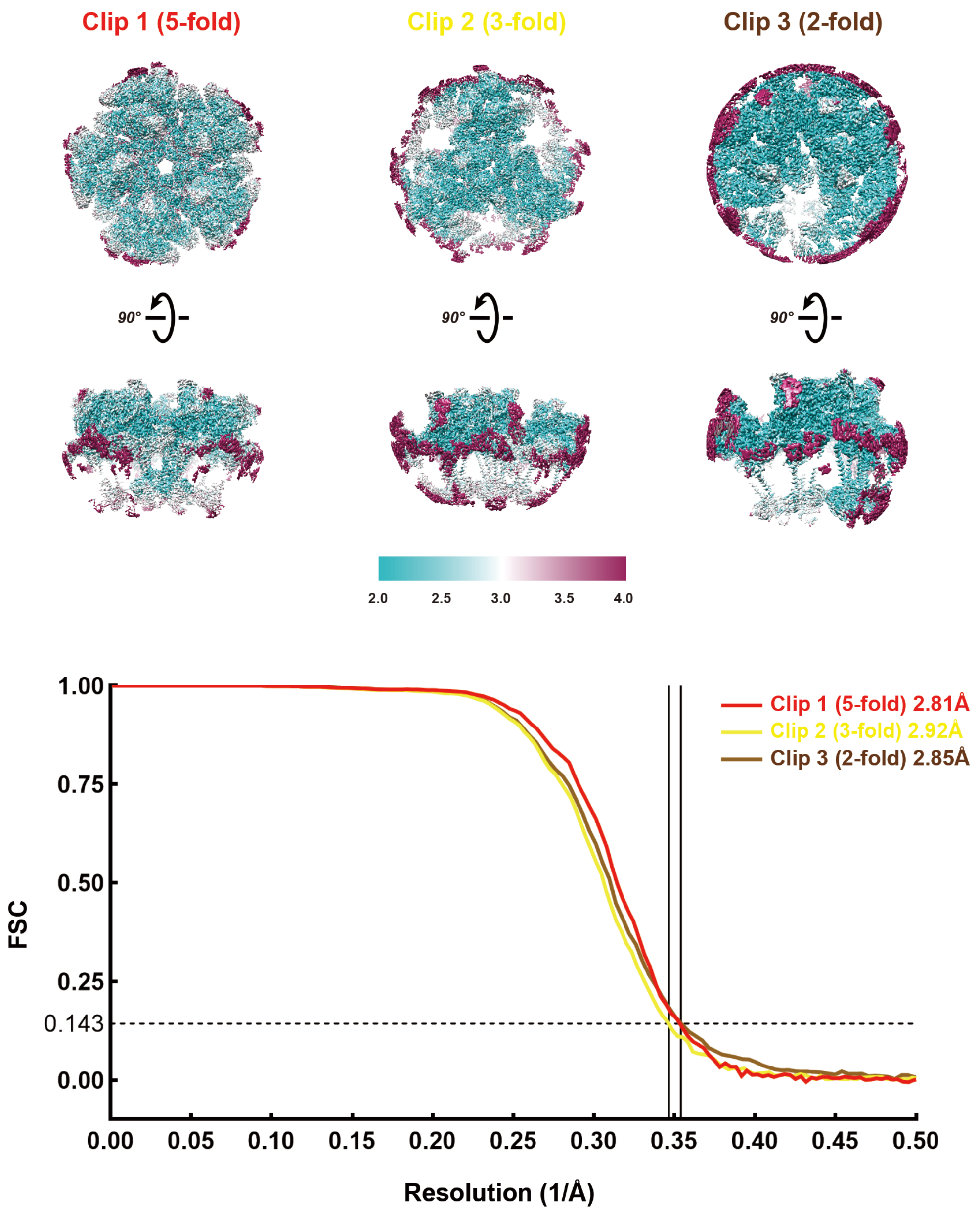

Supplementary Figure S10. Local resolution of cryo-EM block-based reconstructions of GETV.

Block-based reconstructions of GETV, colored according to local resolution. The resolution varies from 2.0 Å (sky blue) through 3.0 Å (light grey) to 4.0 Å (violet-red). Fourier shell correlation (FSC) curve of the final reconstructions indicating an average resolution of 2.81 Å, 2.92 Å, and 2.84 Å for the Clip 1 (5-fold symmetry), Clip 2 (3-fold), and Clip 3 (2-fold), respectively, according to the gold-standard criterion (FSC=0.143).

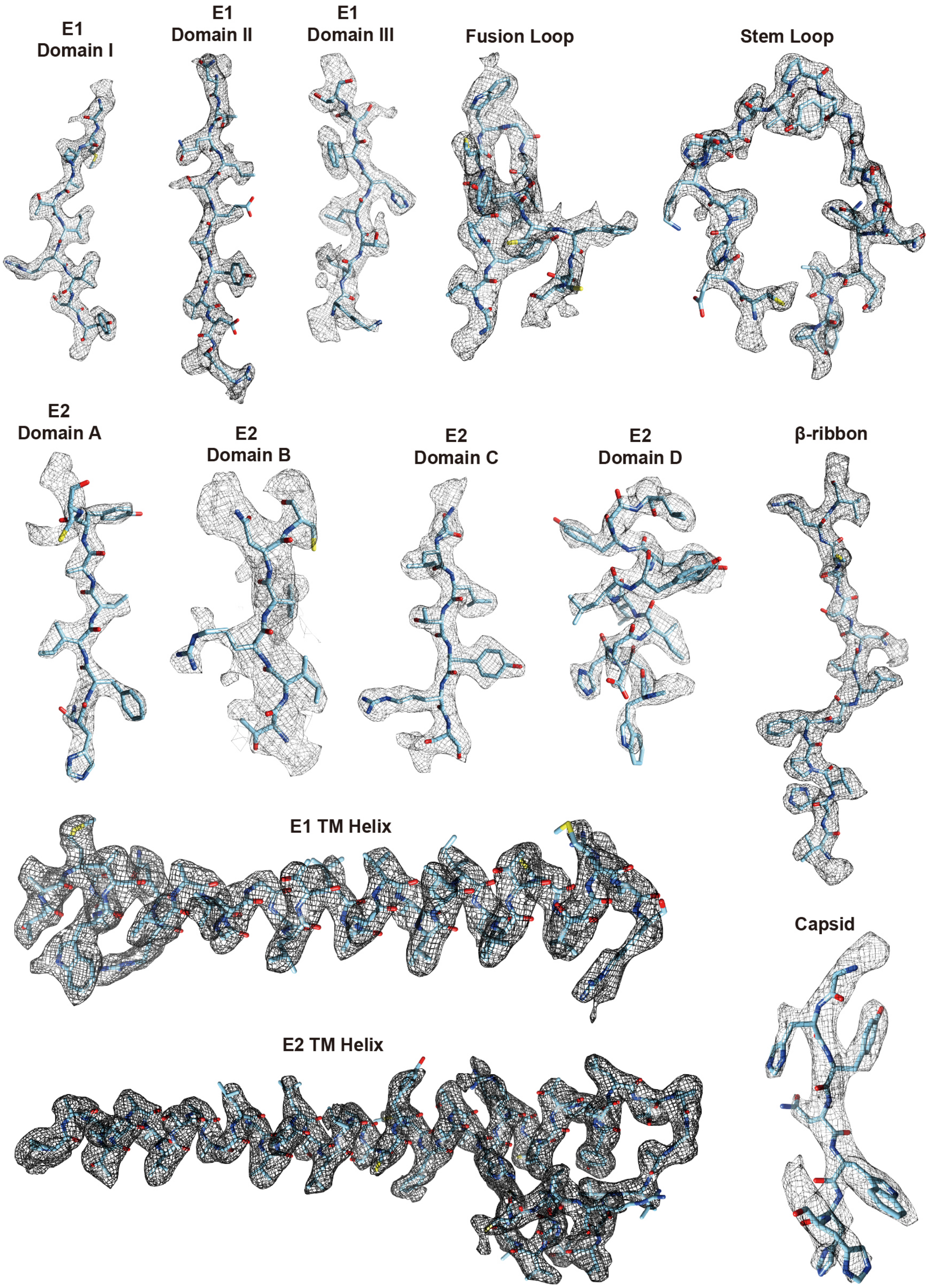

Supplementary Figure S11. Representative density and atomic models of each domain of E1-E2-capsid heterotrimer.

E1 Domain I, residues A128-Y137; E1 Domain II, residues N100-R110; E1domainIII, residues K351-T358; Fusion loop, residues V84-T98, Stem-loop, residues C380-D401; E1 TM Helix, residues T405-R438; E2 Domain A, residues A92-H99; E2 Domain B, residues T196-C201; E2 Domain C, residues T292-S298; E2 Domain D, residues W350-L361; E2 β-ribbon, K253-V267; E2 TM Helix, residues Y362-R419; Capsid, residues E191-H198.

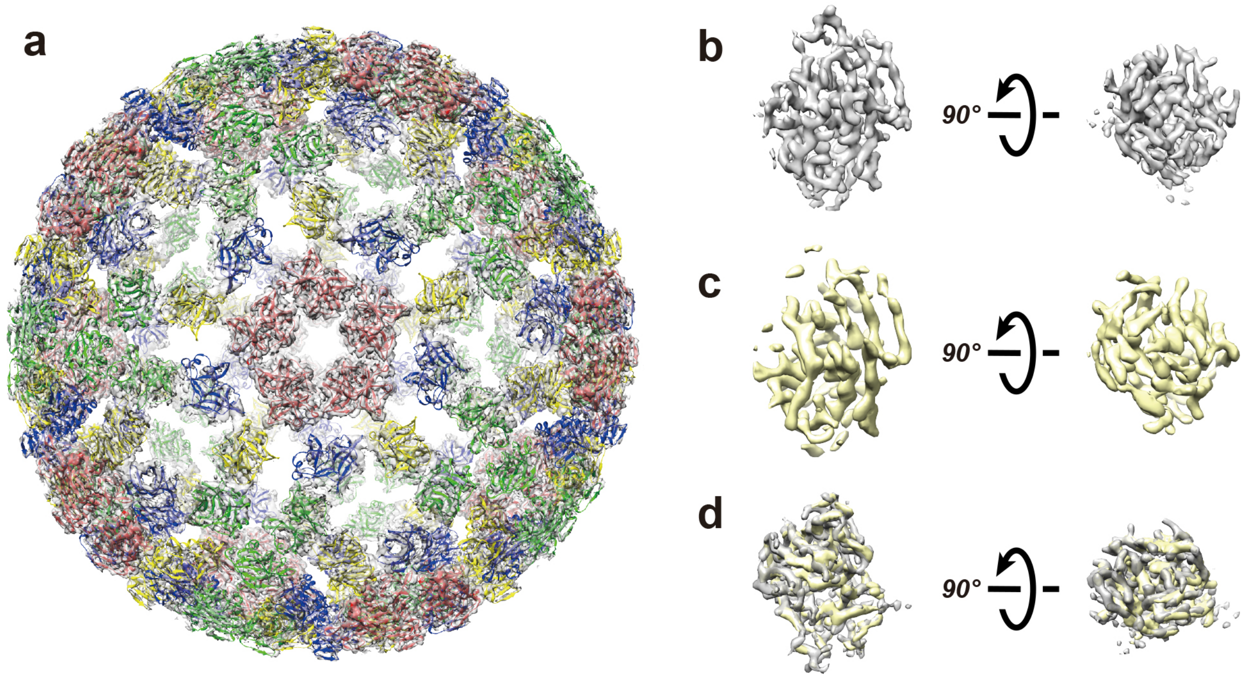

Supplementary Figure S12. Density maps of the capsid.

a, Density map of icosahedral capsid and atomic models. The pentamer capsids models are colored by red, and the other three hexamer capsids models are colored by green, yellow and blue separately. **b**, Density map of the pentamer capsid, **c**, Hexamer capsid, and **d**, Superpose map of pentamer capsid and hexamer capsid. The correlation value is 0.92.

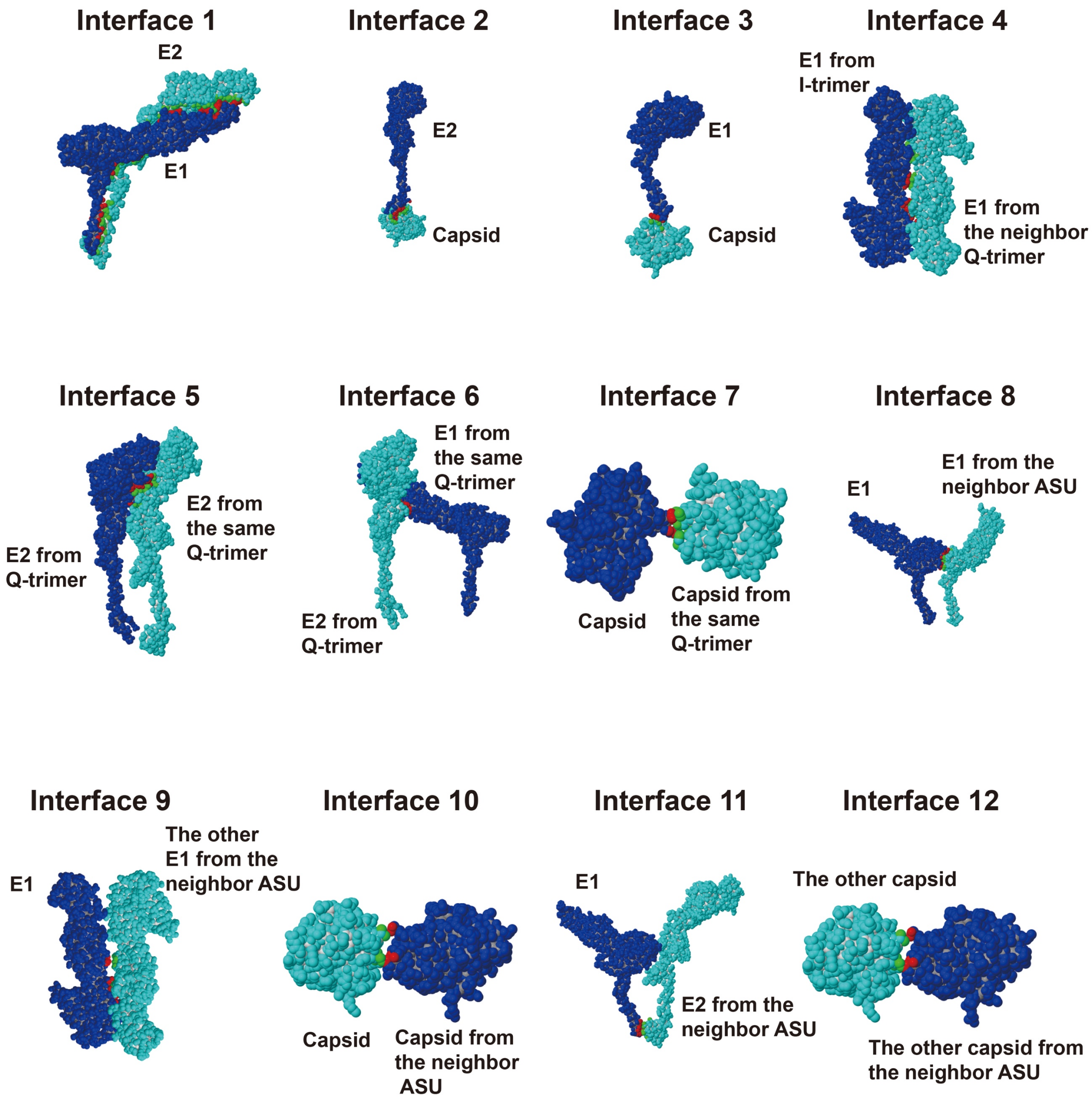

Supplementary Figure S13. Interacting regions between GETV structural proteins.

The interface areas were analyzed by using the PDBePISA server (https://www.ebi.ac.uk/pdbe/prot_int/pistart.html). The structures were rendered as space-fill. The interaction areas are colored in red or green. The relevant protein domains are colored in blue or cyan and labeled on the side. Interface areas, the number of hydrogen bonds (N_{HB}) and salt-bridges (N_{SB}) are listed in Supplementary Table S2.

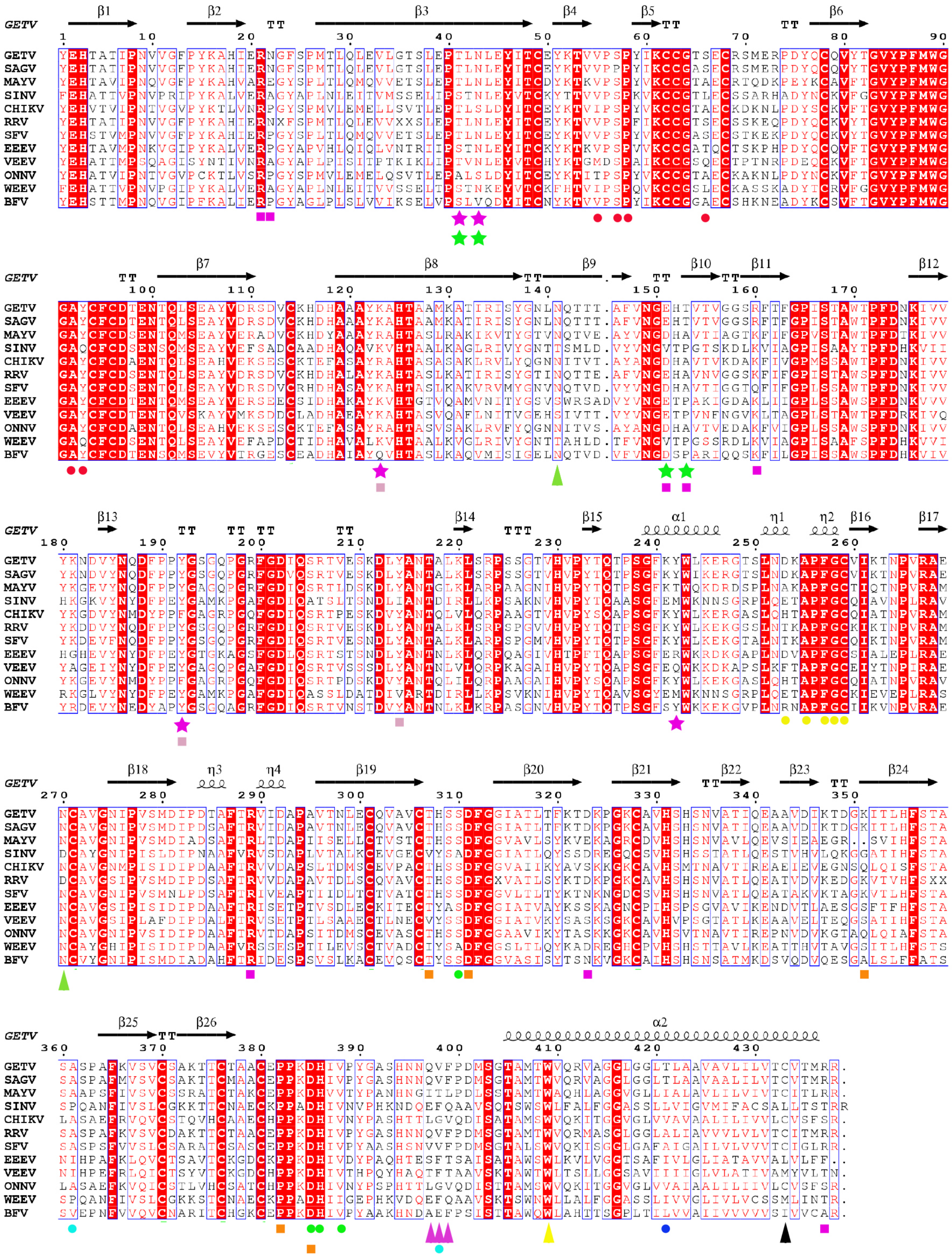

Supplementary Figure S14. Multiple sequence alignment and secondary structural elements of alphaviruses E1 protein.

Sequence alignment of E1 proteins from representative members of alphaviruses. The secondary structure elements are displayed above the sequences. Fully conserved residues and similar residues are shaded and shown in red. Sequences of structural polyprotein are from GETV (Getah virus, this study, ASA40294.1), SAGV (Sagiyama virus, BAA92847.1), MAYV (Mayaro virus, AZM66144.1), SINV (Sindbis virus, AYM45056.1), CHIKV (Chikungunya virus, NP_690589.2), RRV (Ross River virus, NP_062880.1), SFV (Semliki Forest virus, NP_463458.1), EEEV (Eastern equine encephalitis virus, NP_632022.1), VEEV (Venezuelan equine encephalitis virus, NP_040824.1), ONNV (O'nyong nyong virus, NP_041255.1), WEEV (Western equine encephalitis virus, NP_640331.1), and BFV (Barmah Forest virus, NP_054024.1). N-glycosylation sites and S-acylation sites are marked by green and black triangles. Magenta and yellow triangles represent residues that interact with cholesterol and DOPC respectively. The colored dots represent residues that interact between E1 and E2, or E2 and capsid. Dots' colors are the same as the borders of Figure 2b-2e. The colored stars represent residues that interact within the ASU. Stars' colors are the same as the corresponding residues of Figure 3b-3f. The colored squares represent residues that interact between two Q-trimers. Squares' colors are the same as the corresponding residues of Figure 4b-4g.

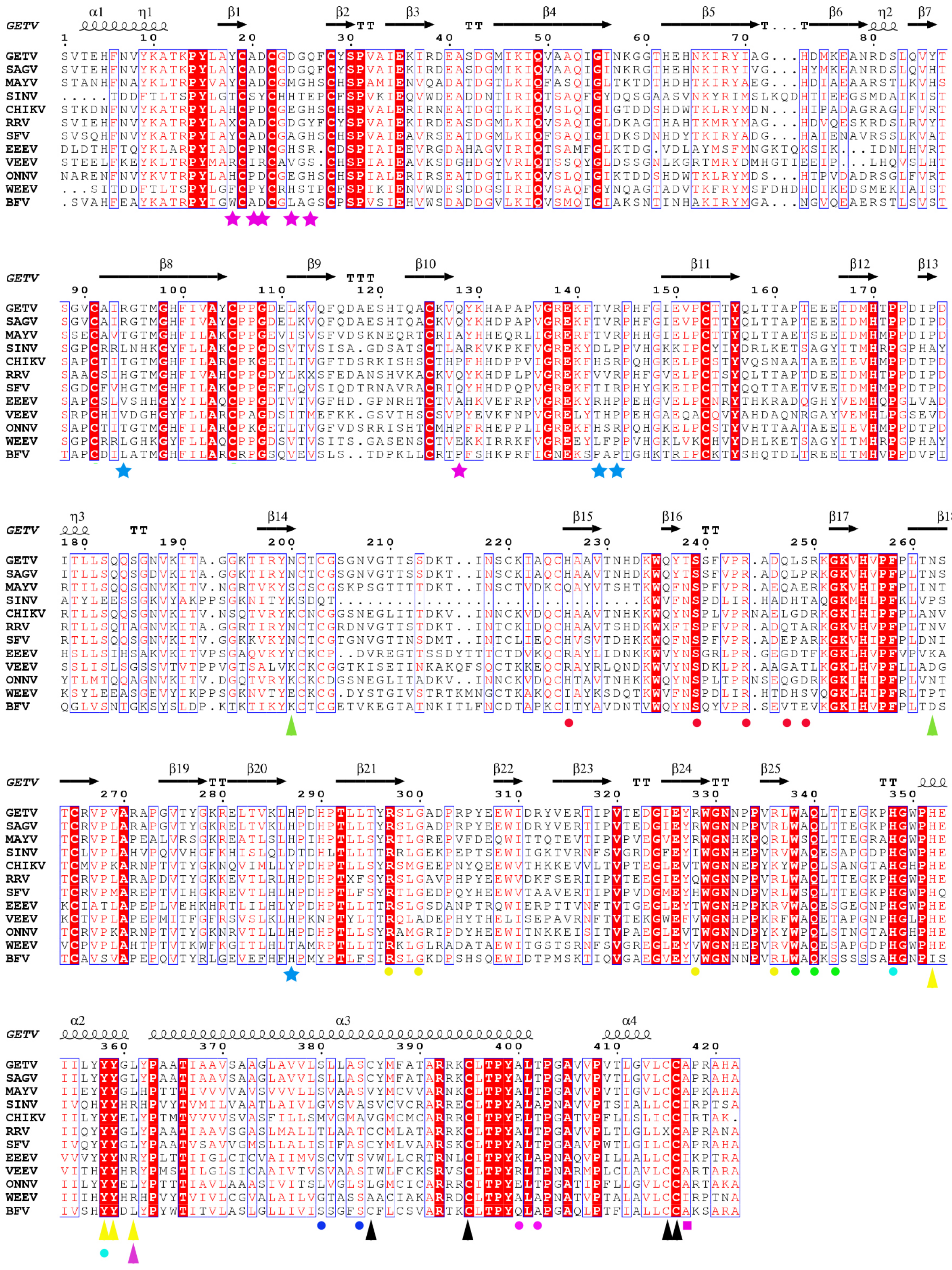

Supplementary Figure S15. Multiple sequences alignment and secondary structural elements of alphaviruses E2 protein.

Sequence alignment of the E2 proteins from representative members of alphaviruses. The secondary structure elements are displayed above the sequences. Fully conserved residues and similar residues are shaded and shown in red. Structural polyprotein sequences and the markers for key residues are the same as in Supplementary Figure S14.

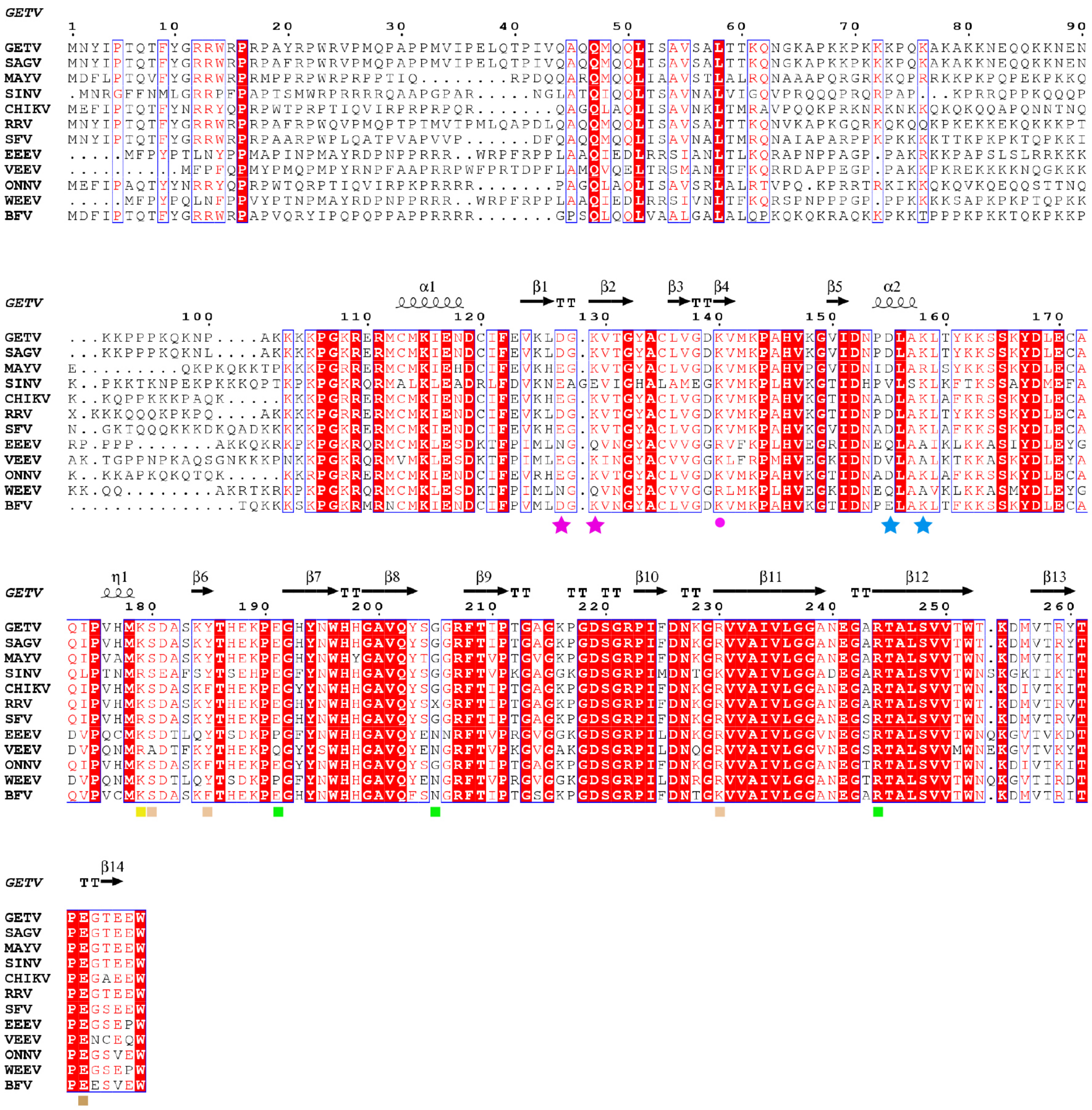

Supplementary Figure S16. Multiple sequences alignment and secondary structural elements of alphaviruses capsid protein.

Sequence alignment of capsid proteins from representative members of alphaviruses. The secondary structure elements are displayed above the sequences. Fully conserved residues and similar residues are shaded and shown in red. Structural polyprotein sequences and the markers for key residues are the same as in Supplementary Figure S14.

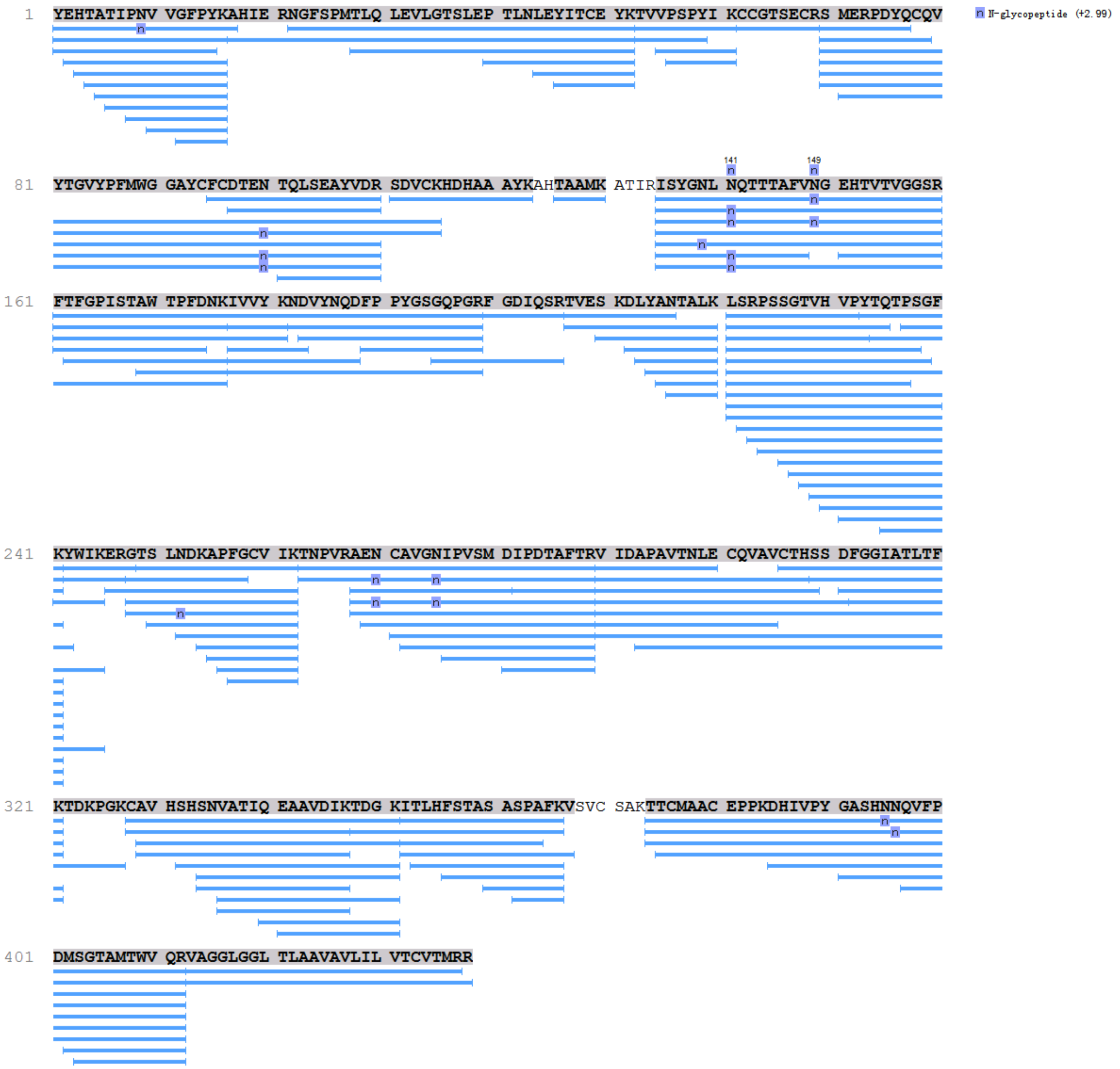

Supplementary Figure S17. Mass spectrometry of the N-glycosylation sites in the GETV E1 protein.

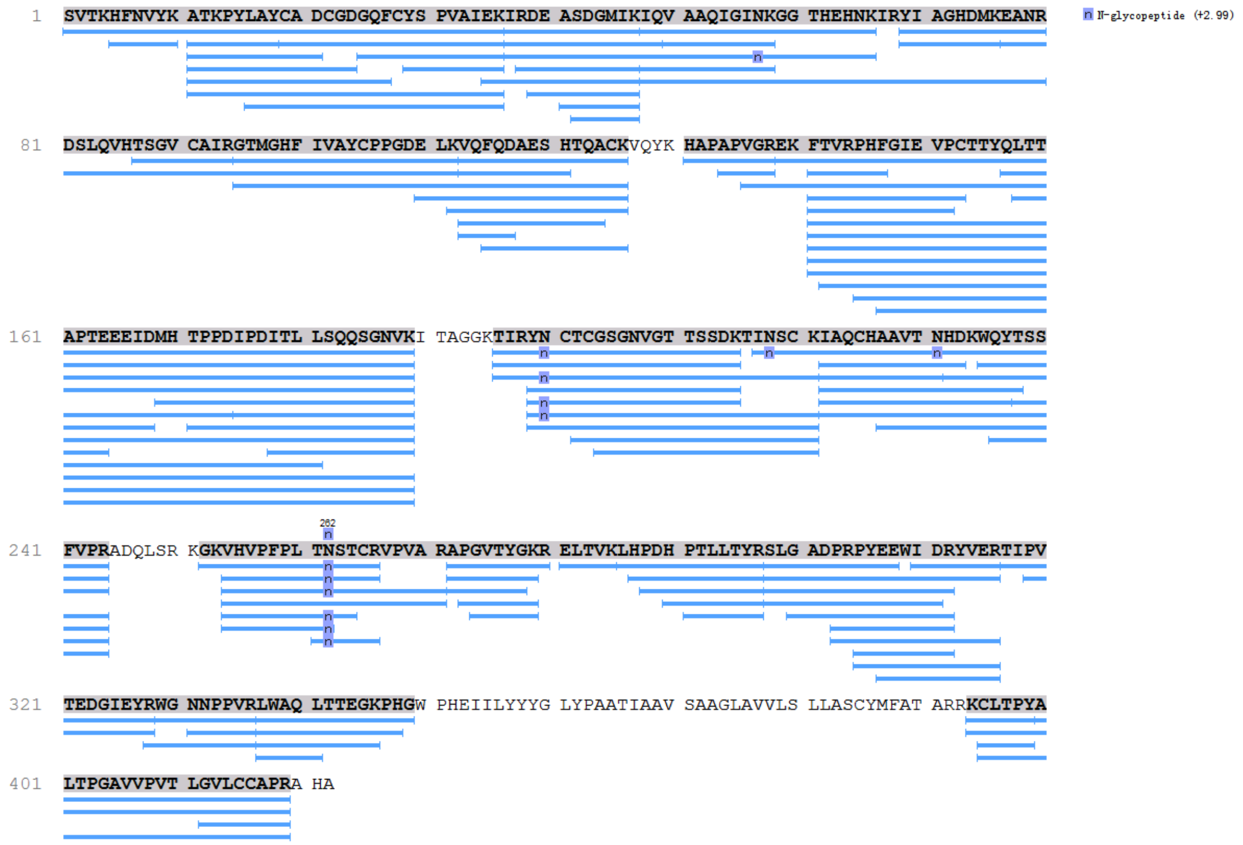

Supplementary Figure S18. Mass spectrometry of the N-glycosylation sites in the GETV E2 protein.

a

E1 N141

Virus	Sequence
GETV	RISYGNLNQTTTAFV
SINV	RIVYGNTTSMLDVYV
MAYV	KVTYGTVNQTVEAYV
CHIKV	RVLYQGNNITVTAYA
VEEV	NITVGEHSIVTTVYV

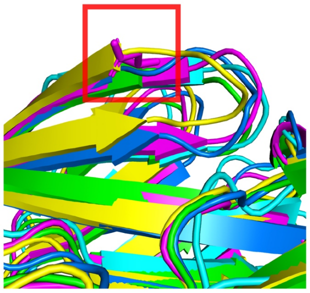

b

E1 N270

Virus	Sequence
GETV	TNPVRAENCAVGNIP
SINV	VNPLRAVDCAYGNIP
MAYV	TNPVRAMNCAVGNIP
CHIKV	TNPVRAMNCAVGNMP
VEEV	TNPIRAENCAVGSIP

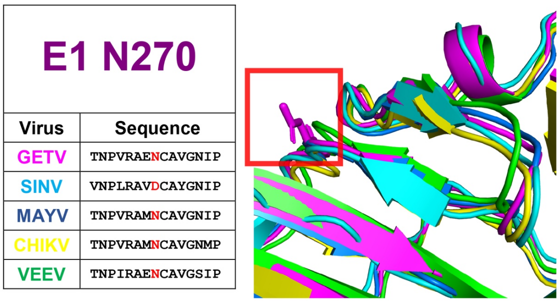

c

E2 N200

Virus	Sequence
GETV	GGKTIRYNCTCGSGN
SINV	SGKNITYKSDQTKWV
MAYV	NGRTVKYSCSCGSKP
CHIKV	NSQTVRYKCNCGGSN
VEEV	VGTSALVKCKCGGTK

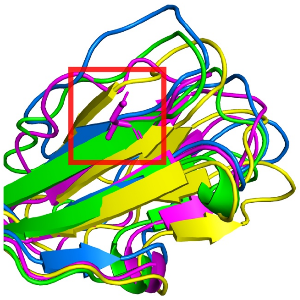

d

E2 N262

Virus	Sequence
GETV	HVPFPLTNSTCRVPV
SINV	HLPFKLVPSTCLVPL
MAYV	HTPFPLINTTCRVPL
CHIKV	HIPFPLANVTCMVPK
VEEV	HVPFLLADGKCTVPL

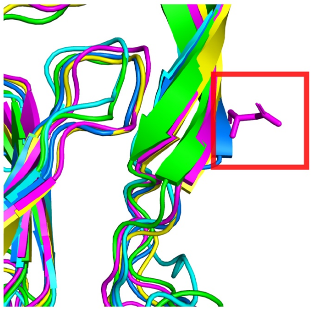

e

E1 S66

Virus	Sequence
GETV	YIKCCGTSECRSMER
SINV	KVKCCGTLECSSARH
MAYV	YVKCCGTAECRTQDK
CHIKV	YVKCCGTAECKDKNL
VEEV	AIKCCGSQECTPTNR

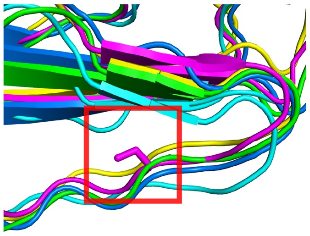

f

E2 T155

Virus	Sequence
GETV	GIEVPCTTYQLTTAP
SINV	GKKIPCYIYDRLKET
MAYV	GIELPCTYQLTTTAE
CHIKV	GKELPCSTYVQSNAA
VEEV	GAEQACQVYAHDAQN

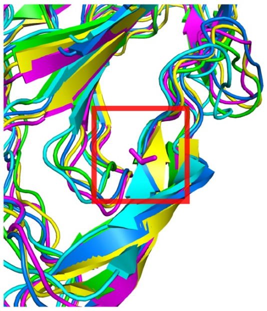

g

E2 T264

Virus	Sequence
GETV	PFPLTNSTCRVPVAR
SINV	PFKLVPSTCLVPLAH
MAYV	PFPLINTTCRVPLAP
CHIKV	PFPLANVTCMVPKAR
VEEV	PFLLADGKCTVPLAP

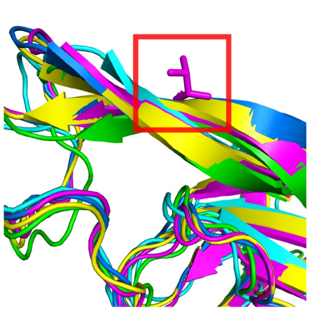

h

E1 N335

Virus	Sequence
GETV	CAVHSHSNVATIQEA
SINV	CSVHSHSSTATLQES
MAYV	CDVHSHSNVAVLQEV
CHIKV	CAVHSMTNAVTIREA
VEEV	CAVHVPSGTATLKEA

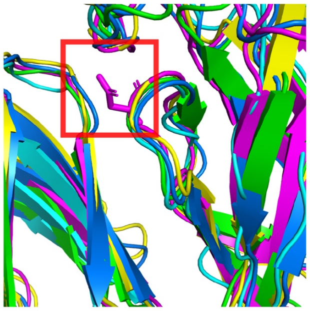

Supplementary Figure S19. Conserved glycosylation sites in alphaviruses.

a-h, Structural differences among alphaviruses at the eight glycosylation sites. Tables on the left show amino acid sequences around the sites, with glycosylation residues

highlighted in red. Figures on the right display the five atomic structures of alphaviruses aligned to the GETV atomic model. The GETV model is colored in magenta, the SINV model in cyan (PDB ID: 6IMM), the MAYV model in marine (PDB ID: 7KO8), the CHIKV model in yellow (PDB ID: 6NK5), and the VEEV model in green (PDB ID: 3J0C).

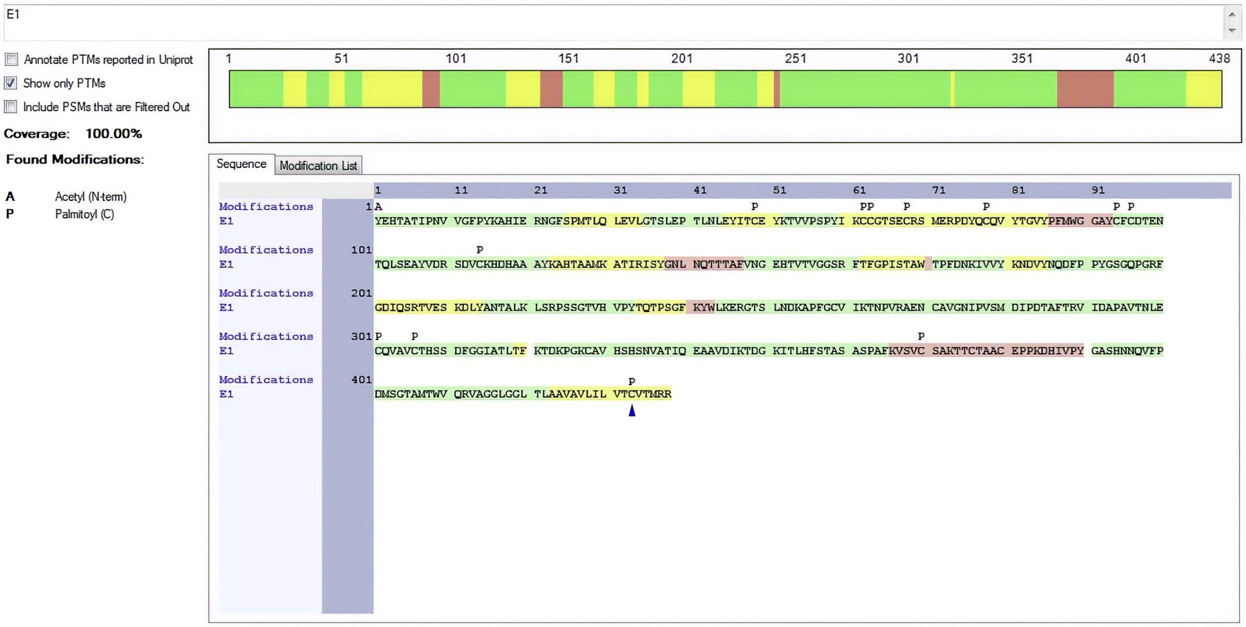

Supplementary Figure S20. Mass spectrometry of the S-acylation sites in the GETV E1 protein.

The protein sequence coverage bar graphically represents the protein sequence. The colored areas represent portions of the protein sequence that match peptides. The protein sequence coverage bar is colored to indicate the confidence of the peptide sequence identification: green, high-confidence peptides; yellow, medium-confidence peptides; pink, low-confidence peptides. Single-letter abbreviation “P” above the sequence indicates the identified palmitoyl sites by mass spectrometry, and the blue arrowhead points to the position of the C433 palmitoylation site identified in the cryo-EM density map.

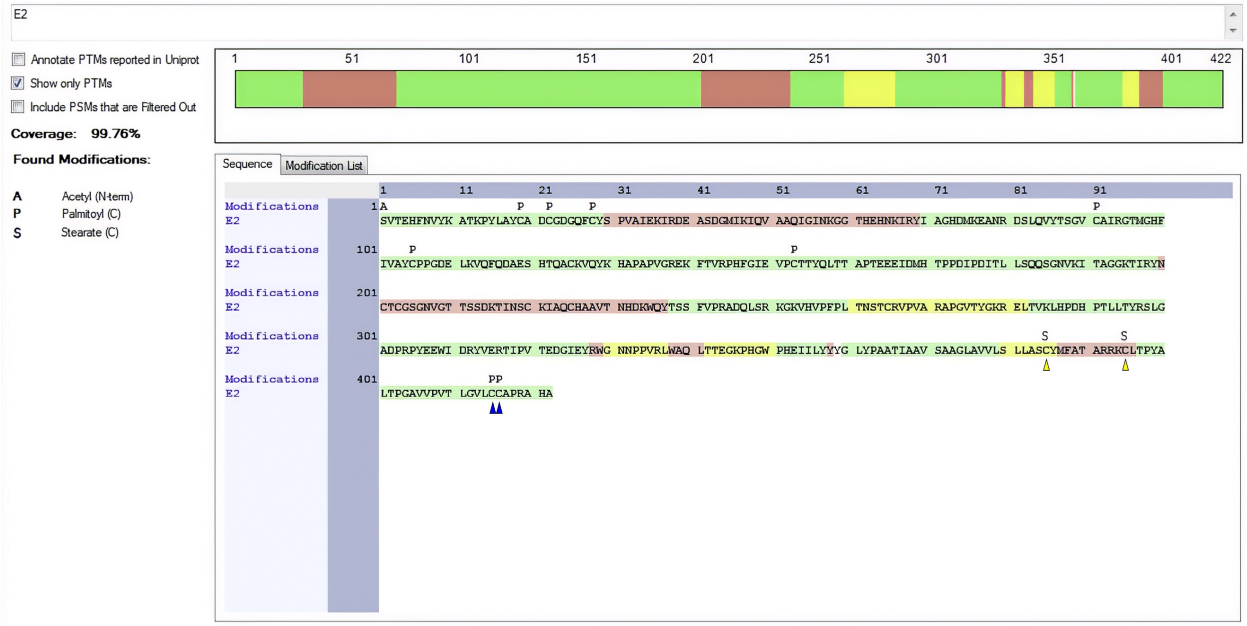

Supplementary Figure S21. Mass spectrometry of the S-acylation sites in the GETV E2 protein.

Single-letters abbreviation “P” and “S” above the sequence indicate the identified palmitoyl or stearate sites by mass spectrometry, and blue and yellow arrowheads point to the positions of C385 and C395 stearate-acylation sites and C415 and C416 palmitoylation sites identified in the cryo-EM density map.

E2 C385

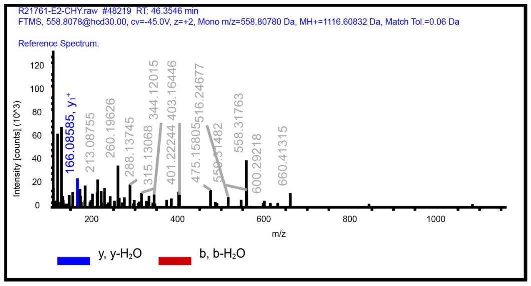

#1	b⁺	b²⁺	Seq.	y⁺	y²⁺	#2
1	114.09134	57.54931	L			7
2	185.12845	93.06787	A	1003.52426	502.26577	6
3	272.16048	136.58388	S	932.48715	466.74721	5
4	641.43062	321.21895	C-Stearate	845.45512	423.2312	4
5	804.49395	402.75061	Y	476.18498	238.59613	3
6	951.52935	476.26831	M-Oxidation	313.12165	157.06447	2
7			F	166.08626	83.54677	1

E2 C395

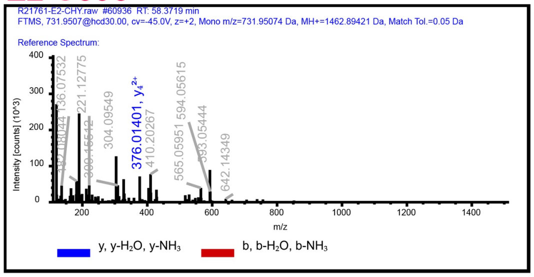

#1	b⁺	b²⁺	Seq.	y⁺	y²⁺	#2
1	132.04776	66.52752	M			10
2	279.11618	140.06173	F	1331.85955	666.43341	9
3	350.15329	175.58028	A	1184.79113	592.8992	8
4	451.20097	226.10412	T	1113.75402	557.38065	7
5	522.23808	261.62268	A	1012.70634	506.85681	6
6	678.33919	339.67323	R	941.66923	471.33825	5
7	834.4403	417.72379	R	785.56811	376.01401	4
8	962.53527	481.77127	K	629.467	315.23714	3
9	1331.8054	666.40634	C-Stearate	501.37204	251.18966	2
10			L	132.10191	66.55459	1

E2 C415 E2 C416

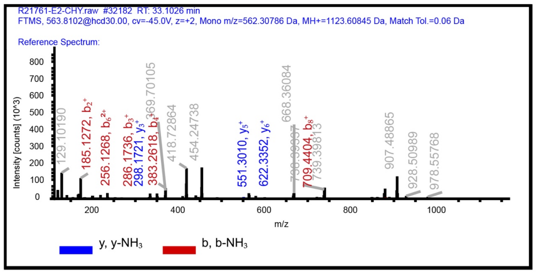

#1	b⁺	b²⁺	Seq.	y⁺	y²⁺	#2
1	72.0449	36.5225	A			23
2	185.1272	93.0645	L	2621.6265	1311.3132	22
3	286.1736	143.5883	T	2508.5422	1254.7711	21
4	383.2618	192.1147	P	2407.4946	1204.2473	20
5	440.2509	220.6254	G	2310.4419	1155.7209	19
6	511.288	256.1268	A	2253.4204	1127.2102	18
7	610.3564	305.6782	V	2182.3833	1091.6917	17
8	709.4404	355.2124	V	2083.3149	1042.1575	16
9	806.4776	403.7388	P	1984.2285	992.6024	15
10	905.546	453.273	V	1887.1937	944.0969	14
11	1006.5937	503.7968	T	1788.1099	894.5627	13
12	1119.6777	560.3389	L	1687.0723	844.0388	12
13	1176.6992	588.8496	G	1573.9812	787.4968	11
14	1275.7676	638.3838	V	1516.9534	758.9861	10
15	1388.8517	694.9258	L	1417.8982	709.4404	9
16	1730.0906	865.5453	C-Plamitoyol	1304.8132	652.9099	8
17	2071.3293	1036.1647	C-Plamitoyol	963.5817	482.2904	7
18	2142.3665	1071.6832	A	622.3352	311.671	6
19	2239.4192	1120.2096	P	551.301	276.1524	5
20	2395.5203	1198.2601	R	454.2521	227.626	4
21	2466.5574	1233.7787	A	298.1721	149.5755	3
22	2603.6165	1302.3082	H	227.1139	114.0569	2
23			A	90.0549	45.5275	1

E1 C433

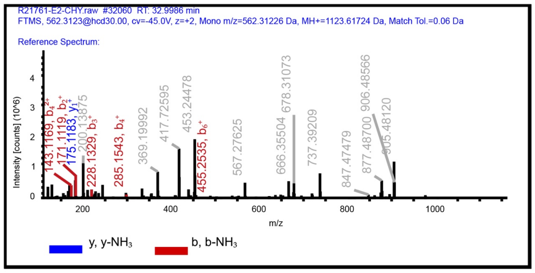

#1	b⁺	b²⁺	Seq.	y⁺	y²⁺	#2
1	100.0762	50.5381	V			26
2	171.1119	86.0567	A	2709.6497	1355.3248	25
3	228.1329	114.5614	G	2638.6125	1319.8063	24
4	285.1543	143.1169	G	2581.5911	1291.2955	23
5	398.2403	199.6202	L	2524.5696	1262.7848	22
6	455.2535	228.1329	G	2411.4768	1206.2356	21
7	512.2833	256.6416	G	2354.4307	1177.7169	20
8	625.3673	313.1837	L	2297.4141	1149.2213	19
9	726.415	363.7075	T	2184.3586	1092.671	18
10	839.499	420.2495	L	2083.3108	1042.1554	17
11	910.5361	455.7681	A	1970.2205	985.6141	16
12	981.5732	491.2866	A	1899.1897	950.0835	15
13	1080.6417	540.8209	V	1828.1526	914.5763	14
14	1151.6788	576.3394	A	1729.0842	865.0421	13
15	1250.7472	625.8736	V	1658.0471	829.5236	12
16	1363.8313	682.4156	L	1558.9786	779.9893	11
17	1476.9153	738.9576	I	1445.8947	723.4473	10
18	1589.9994	795.4997	L	1332.8105	666.9053	9
19	1689.0677	845.0339	V	1219.7266	610.3633	8
20	1790.1155	895.5577	T	1120.6581	560.829	7
21	2131.3542	1066.1771	C-Plamitoyol	1019.6104	510.3052	6
22	2230.4229	1115.7114	V	678.3715	339.6858	5
23	2331.4705	1166.2352	T	579.3031	290.1516	4
24	2478.5059	1239.7529	M-Oxidation	478.2554	239.6277	3
25	2634.6069	1317.8035	R	331.22	166.11	2
26			R	175.1183	88.0595	1

Supplementary Figure S22. MS spectra and table of mass shifts of S-acylation by LC-MS/MS.

The left panel is the mass spectra of the peptide containing the S-acylation site, of which the abscissa is the mass-charge ratio (M/Z) and the ordinate is the ion peak intensity. The red is the detected y ion and the blue is the detected b ion. After the peptide fragments of proteins are broken under the action of energy, b ions and y ions will be produced, in which the N-terminal fragment is the b ion, and the C-terminal fragment is the y ion. The black peaks are the ion peaks detected by the instrument. The blue and red peaks are the ion peaks detected by the instrument and matched to the theoretical fragment ion peak generated by the fragmentation of the peptide. The blue and red peaks are the ion peaks detected by the instrument and matched with the black peak. The b ions and y ions correspond to each other, and the corresponding relationship is shown in the right panel. S-acylation modification occurs on CKST amino acids, for which the molecular weight change after modification is 238.23/266.26 Da (palmitic acid/stearic acid). The MS data were analyzed for protein modification using Proteome Discoverer 2.4.

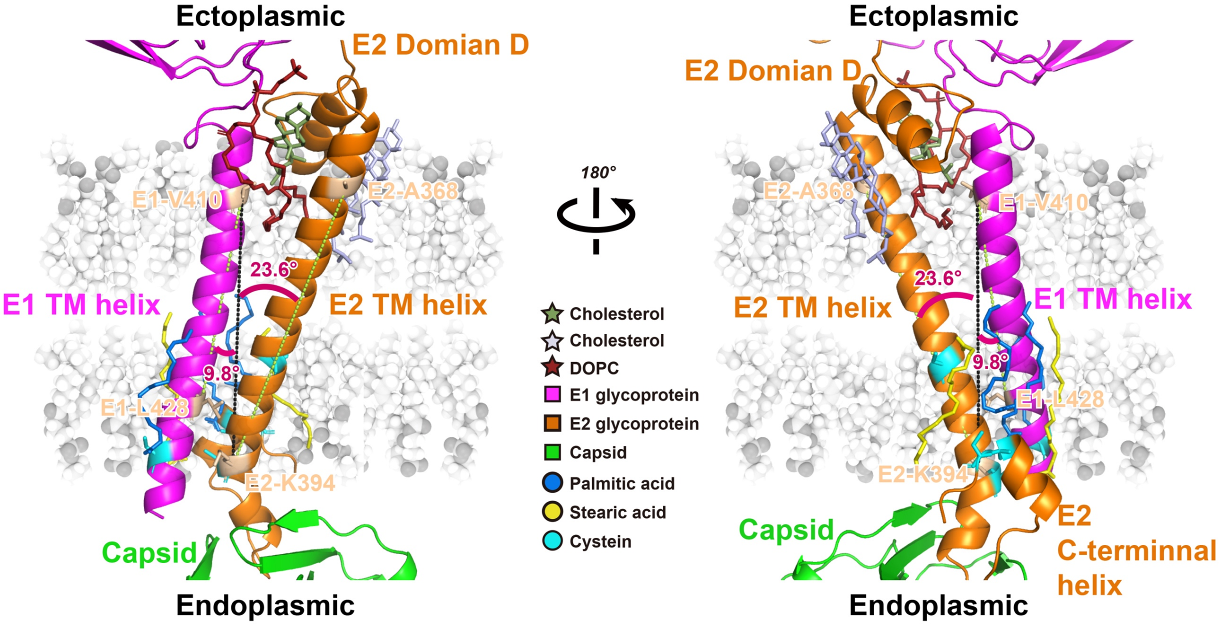

Supplementary Figure S23. Transmembrane helices of E1 and E2.

The black dash line between Cα of E1-V410 and E2-K394 is perpendicular to the lipid bilayer. The lime green dashes lines between Cα of E1-V410 and E1-L428, E2-A368 and E2-K394 are parallel to E1 and E2 helix, respectively. The angles between E1 and lipid bilayer, and the E2 and lipid bilayer are 9.8° and 23.6°, respectively. Residues E1-V410, E1-L428, E2-A368 and E2-K394 are shown as sticks and colored in wheat. Cholesterol, DOPC, cysteines, palmitic acids, and stearic acids are shown as sticks.

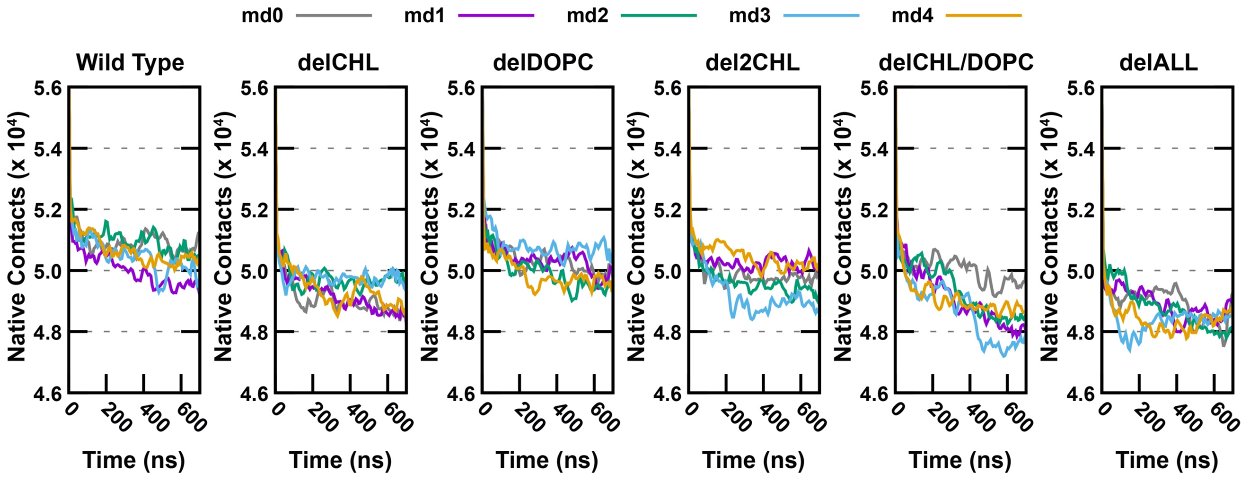

Supplementary Figure S24. Time evolutions from MD simulation analyses for the native contacts within the E1-E2 complex.

Time evolutions from MD simulation analysis for the number of native contacts of E1 (295-435) and E2 (269-422) for systems “Wild Type”, “delCHL”, “delDOPC”, “del2CHL”, “delCHL/DOPC”, and “delALL”. All curves were smoothed with the Bezier method and implemented in gnuplot.

a

Virus	Protein	Sequence
GETV	E1	NNQVFPDMSGTAMTWVQRVAGG
	E2	PHEIILYYYGLYPAATIAAVSAA
SINV	E1	DQEFQAAVSQTSWSWLFALFGG
	E2	PHEIVQHYYHRHPVYTVMILVAA
MAYV	E1	NGITLPDLSSTAMTWAQHLAGG
	E2	PHEIIEYYYGLHPTTTIVVVVAV
VEEV	E1	AQTFTAAVSKTAWTWLTSLLGG
	E2	PHEVITHYYHRYPMSTILGLSIC

b

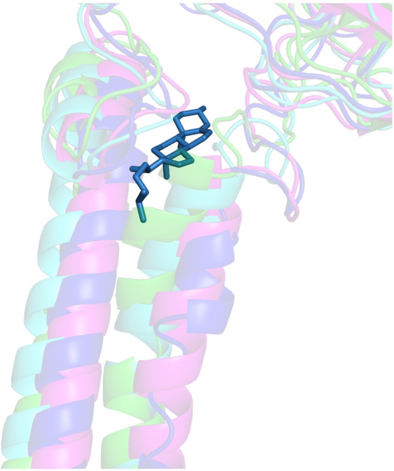

c **d** **e** **f**

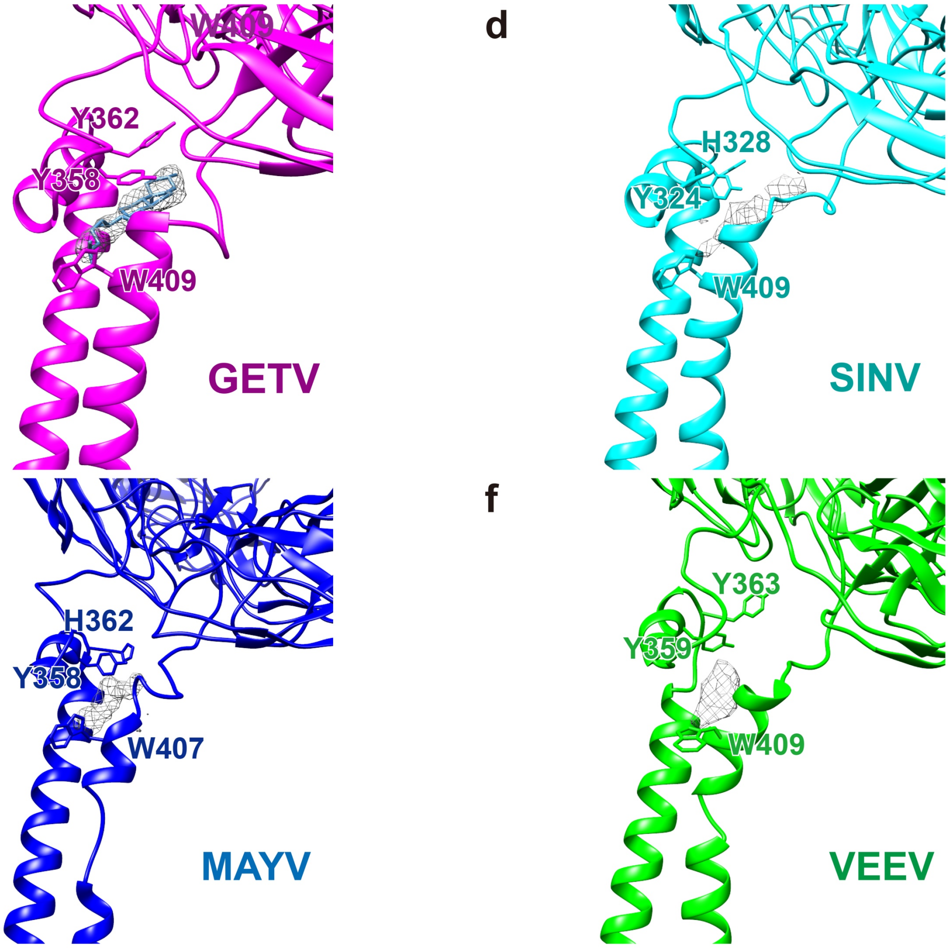

Supplementary Figure S25. Comparison of the hydrophobic pocket structures between GETV and other alphaviruses.

a, Table showing amino acid sequences of E1 and E2 helixes from GETV, SINV, MAYV, and VEEV. Residues involving cholesterol binding in the hydrophobic pocket are colored in red. **b**, Atomic models of GETV, SINV, MAYV, and VEEV are superimposed and

colored in magenta, cyan, marine, and green, respectively. **c** – **f**, Zoom-in views of the hydrophobic pocket in GETV, SINV, MAYV, and VEEV, with densities in the hydrophobic pocket shown as a mesh.

Supplementary Table S1. Cryo-EM data collection and processing, block-based reconstruction, model building, and refinement statistics.

**Data Collection & Processing**			
Microscope	FEI Titan Krios		
Camera	Gatan K3		
Magnification	105K		
Voltage(kV)	300		
Total dose ($e^{-}/Å^2$)	40		
Defocus range (μm)	0.8-1.5		
Pixel size (Å/pixel)	0.83		
Symmetry imposed	I3		
Movies (total)	16,894		
Initial particles images	171,059		
Final particles images	100,226		
**Block-based Reconstruction**			
Block items	Clip1(5-fold)	Clip2(3-fold)	Clip3(2-fold)
Block symmetry imposed	C1		
Initial block images	6,013,560		
Final block images	2,041,957	2,446,428	2,889,370
Final resolution (Å)	2.81	2.92	2.85
**Asymmetric Unit Model Building & Refinement**			
Initial model used (PDB code)	3J0C		
Composition			
Atoms	32,620		
Residues	4060 amino acids		
Water	0		
Ligand	STE, PLM, PCW, CLR, NAG, BMA, MAN		
Bonds (RMSD)			
Length (Å) (# > 4σ)	0.036		
Angles (°) (# > 4σ)	2.021		
Clash score	2.85		
Rotamer outliers (%)	1.44		
Ramachandran plot (%)			
Outliers	0.30		
Allowed	2.80		
Favored	96.90		

Supplementary Table S2. Interaction areas between GETV structural proteins.

	Interaction area, (Å^2)	N_{HB}	N_{SB}
**Interface 1**	3319.1	34	2
**Interface 2**	568.9	5	0
**Interface 3**	107.0	1	1
**Interface 4**	594.9	5	1
**Interface 5**	523.2	9	1
**Interface 6**	444.4	2	0
**Interface 7**	163.6	2	2
**Interface 8**	599.1	4	3
**Interface 9**	367.1	5	1
**Interface 10**	341.0	2	1
**Interface 11**	185.6	1	0
**Interface 12**	91.7	1	2

interface areas, the number of hydrogen bonds (N_{HB}) and salt-bridges (N_{SB}) are listed in the table. The serial number of interfaces are illustrated in Figure S13.

Supplementary Table S3. Protein-protein interactions in GETV and other alphaviruses

GETV				Other Alphaviruses				
**In the E1-E2-Capsid heterotrimer**								
**Figure**	**Interaction**	**E2**	**E1**	**Alphavirus**	**Interaction**	**E2**	**E1**	**References**
Fig. 2B	HB	H226	A92	CHIKV	HB	H226	A92	Voss et al., 2010 [5]
	HB	S239	S57	CHIKV	HB	S239	S57	Voss et al., 2010
	HB	R244	P58	CHIKV	HB	R244	P58	Voss et al., 2010
	HB	Q247	S66					
	HB	S249	V55					
Fig. 2C	HB	R297	A255	MAYV	HB	R297	A255, N252	Ribeiro-Filho et al., 2021 [6]
	HB	G300	F257	CHIKV	HB	G301	F257	Voss et al., 2010
	SB	R328	D253					
	HB	R336	G258	CHIKV	VDM	K337	I387, N389	Voss et al., 2010
Fig. 2D	HB	W338	V388	CHIKV	HB	Y339	V388	Voss et al., 2010
	HB	Q340	S310, D385, H386	CHIKV	SB	Q341	S310, D385, H386	Voss et al., 2010
	HB	T342	H386					
Fig. 2E	HB	H348	A361	MAYV	HB	H348	T403	Ribeiro-Filho et al., 2021
	HB	Y358	V398					
Fig. 2F	HB	S380	T421					
	HB	S384	T421					
**Figure**	**Interaction**	**E2**	**Capsid**	**Alphavirus**	**Interaction**	**E2**	**Capsid**	**References**
Fig. 2G	HB	A400	K140					
	HB	T402	K140					
**In the asymmetric unit**								
**Figure**	**Interaction**	**E2**	**E2**	**Alphavirus**	**Interaction**	**E2**	**E2**	**References**
Fig. 3B	HB	Y18	R144	CHIKV	VDW	H18	H142, S143, R144, P145, Q146	Voss et al., 2010
	HB	A20	R144	CHIKV	VDW	P20	H142, S143, R144,	Voss et al., 2010

							P145, Q146	
	SB	D21	R144	CHIKV	VDW	D21	H142, S143, R144, P145, Q146	Voss et al., 2010
	SB	D24	R94	CHIKV	VDW	E24	H142, S143, R144, P145, Q146	Voss et al., 2010
	HB	Q26	R94	CHIKV	VDW	H26	H142, S143, R144, P145, Q146	Voss et al., 2010
	HB	Q128	T142	CHIKV	VDW	P128	H142, S143, R144, P145, Q146	Voss et al., 2010
	HB	T142	Q128	CHIKV	VDW	H142	H18, P20, D21, E24, G25, H26, S27, E109, T110, T126, P128,	Voss et al., 2010
**Figure**	**Intera ction**	**E2**	**E1**	**Alphavirus**	**Intera ction**	**E2**	**E1**	**References**
Fig. 3C	HB	H287	Y242	CHIKV	VDW	Y288	Q218, S234, Q235, A236, P237,	Voss et al., 2010
**Figure**	**Intera ction**	**E1**	**E1**	**Alphavirus**	**Intera ction**	**E1**	**E1**	**References**
Fig. 3D	SB	K123	E151	CHIKV	VDW	R123	N149, D151, H152	Voss et al., 2010
	HB	Y192	E151, T153	CHIKV	VDW	F192	Y147	Voss et al., 2010
Fig. 3E	HB	T41	N43	SFV	HB	T41	N43	Roussel et al., 2006[7]
	HB	N43’	T41’	SFV	HB	N43	T41	Roussel et al., 2006
**Figure**	**Intera ction**	**Capsid**	**Capsid**	**Alphavirus**	**Intera ction**	**Capsid**	**Capsid**	**References**

Fig. 3F	HB	D120	K151					
	HB	K122	D148					
**Between two asymmetric units**								
**Figure**	**Intera ction**	**E1**	**E1**	**Alphavirus**	**Intera ction**	**E1**	**E1**	**References**
Fig. 4B	HB, SB	E151	K123, Y192	CHIKV	VDW	D151	R123, K176, P191, W192, G193, A194, Y214, R206	Voss et al., 2010
	HB	T153	Y192	CHIKV	VDW	A153	W192, G193, A194	Voss et al., 2010
	HB	R160	Y192					
	HB	R160	Y214					
Fig. 4C	SB	R21	D385	SFV	SB	R21	D284	Roussel et al., 2006
	HB	N22	T307, P382	CHIKV	VDW	P22	T307, H381, P382, P383, R384	Voss et al., 2010
	SB	R289	D311					
Fig. 4D	SB	D323	K351	CHIKV	VDW	S323	V315, I317, K319, E353	Voss et al., 2010
**Figure**	**Intera ction**	**Capsid**	**Capsid**	**Alphavirus**	**Intera ction**	**Capsid**	**Capsid**	**References**
Fig. 4E	HB	S180	R244					
	HB	Y185	G205					
	SB	R230	E191					
Fig. 4F	SB	E263	K179					
**Figure**	**Intera ction**	**E2**	**E1**	**Alphavirus**	**Intera ction**	**E2**	**E1**	**References**
Fig. 4G	HB	A417	R437					

HB, Hydrogen bonds; SB, salt bridges; VDM, van der Waals contact.

Contacts were calculated using PISA[8].

Interactions highlighted in red represent the newly discovered interactions in the present study.

Supplementary Table S4. Glycosylation sites in the E1 and E2 proteins

Protein	Glycosylation Type	Method	Glycosylation Site
E1	O-Glycosylation	NetOGlyc[9] (www.cbs.dtu.dk/services/NetOGlyc/)	S250
		YinOYang[10] (www.cbs.dtu.dk/services/YinOYang/)	T53, **S66**, S104, T132, T144, S222, S226, T234, T288, S297, T368, S377, T435
		Previously reported	SINV S238 (no density)[6]
		**Cryo-EM density (no atomic model)**	**S66**
	N-Glycosylation	NetNGlyc[10] (www.cbs.dtu.dk/services/NetNGlyc/)	N9, N22, N43, N100, N139, **N141,** N149, N175, N182, N186, N216, N252, N264, **N270**, N275, N298, **N335**, N395, N396.
		GlycoMine[11] (glycomine.erc.monash.edu/Lab/GlycoMine/)	N9, N22, N43, N100, N139, **N141,** N149, N175, N182, N186, N216, N252, N264, **N270**, N275, N298, N395, N396.
		**Mass spectrometry**	N9, N100, N139, **N141**, N149, N252, N264, **N270**, N275, N395, N396.
		Previously reported	SINV N139, N245 (density, no atomic model) [12]<br>**CHIKV, MAYV N141 (atomic model)**[5,6]
		**Cryo-EM density (no atomic model)**	**N335**
		**Cryo-EM density (atomic model)**	**N141, N270**
E2	O-Glycosylation	NetOGlyc	T142, T154, **T155**, T159, S160, S163.
		YinOYang	T3, T87, S88, T122, S185, T211, S212, S213, T230, T238, S240, T261, **T264**, T343, T366.
		Previously reported	N/A
		**Cryo-EM density (no atomic model)**	**T155, T264**
	N-Glycosylation	NetNGlyc	N7, N33, N57, N65, N79, N187, **N200**, N207, N218, N231, **N262**, N332.
		GlycoMine	N7, N57, N65, N79, N187, **N200**, N207, N218, N231, **N262**, N331, N332.
		**Mass spectrometry**	N57, N187, **N200**, N218, N231, **N262**.
		Previously reported	**CHIKV N196 (atomic model)**[5]

			**MAYV N262 (atomic model)**[6]<br>SINV N283 (density, no atomic model)[12]
		**Cryo-EM density (atomic model)**	**N200, N262**

Supplementary Table S5. S-acylation sites in the E1 and E2 proteins

Protein	Acylation Type	Method	S-acylation Site
E1	S-acylation	CSS-Palm[13] (www.csspalm.biocuckoo.org/online.php)	C62, C63
		GPS-Palm[14] (gpspalm.biocuckoo.cn/)	C63, C259, **C433**
		Mass spectrometry	C49, C62, C63, C68, C78, C94, C96, C114, C301, C306, C370, **C433**
		Previously reported	BFV C436 (density, no atomic model)[15]
		**Cryo-EM density (atomic model)**	**C433**
	N-acylation, O-acylation	Mass spectrometry	S136, T143
		Previously reported	N/A
		**Cryo-EM density**	**N/A**
E2	S-acylation	CSS-Palm	C19, C22, **C415**, **C416**
		GPS-Palm	**C385**, **C395**, **C415**, **C416**
		Mass spectrometry	C19, C22, C28, C91, C105, C153, **C385**, **C395**, **C415**, **C416**
		Previously reported	BFV C384, C387, C394, C414, C415 (density, no atomic model)[15] VEEV C396, C416, C417 (density, no atomic model)[16]
		**Cryo-EM density (atomic model)**	**C385, C395, C415, C416**
	N-acylation, O-acylation	Mass spectrometry	T130, T159, T171, K195, T196
		Previously reported	N/A
		**Cryo-EM density**	**N/A**

References

1 Kumanomido, T. *et al.* Clinical and virological observations on swine experimentally infected with Getah virus. *Vet Microbiol* **16**, 295-301, doi:10.1016/0378-1135(88)90033-8 (1988).

2 Zhou, F. *et al.* Isolation and phylogenetic analysis of Getah virus from a commercial modified live vaccine against porcine reproductive and respiratory syndrome virus. *Mol Cell Probes* **53**, 101650, doi:10.1016/j.mcp.2020.101650 (2020).

3 Snyder, J. E. *et al.* Functional characterization of the alphavirus TF protein. *J Virol* **87**, 8511-8523, doi:10.1128/jvi.00449-13 (2013).

4 Zheng, S. Q. *et al.* MotionCor2: anisotropic correction of beam-induced motion for improved cryo-electron microscopy. *Nat Methods* **14**, 331-332, doi:10.1038/nmeth.4193 (2017).

5 Voss, J. E. *et al.* Glycoprotein organization of Chikungunya virus particles revealed by X-ray crystallography. *Nature* **468**, 709-712, doi:10.1038/nature09555 (2010).

6 Ribeiro-Filho, H. V. *et al.* Cryo-EM structure of the mature and infective Mayaro virus at 4.4 Å resolution reveals features of arthritogenic alphaviruses. *Nat Commun* **12**, 3038, doi:10.1038/s41467-021-23400-9 (2021).

7 Roussel, A. *et al.* Structure and interactions at the viral surface of the envelope protein E1 of Semliki Forest virus. *Structure* **14**, 75-86, doi:10.1016/j.str.2005.09.014 (2006).

8 Lawrence, M. C. & Colman, P. M. Shape complementarity at protein/protein interfaces. *J Mol Biol* **234**, 946-950, doi:10.1006/jmbi.1993.1648 (1993).

9 Steentoft, C. *et al.* Precision mapping of the human O-GalNAc glycoproteome through SimpleCell technology. *EMBO J* **32**, 1478-1488, doi:10.1038/emboj.2013.79 (2013).

10 Gupta, R. & Brunak, S. Prediction of glycosylation across the human proteome and the correlation to protein function. *Pac Symp Biocomput*, 310-322 (2002).

11 Li, F. *et al.* GlycoMine: a machine learning-based approach for predicting N-, C- and O-linked glycosylation in the human proteome. *Bioinformatics* **31**, 1411-1419, doi:10.1093/bioinformatics/btu852 (2015).

12 Chen, L. *et al.* Implication for alphavirus host-cell entry and assembly indicated by a 3.5Å resolution cryo-EM structure. *Nat Commun* **9**, 5326, doi:10.1038/s41467-018-07704-x (2018).

13 Ren, J. *et al.* CSS-Palm 2.0: an updated software for palmitoylation sites prediction. *Protein Eng Des Sel* **21**, 639-644, doi:10.1093/protein/gzn039 (2008).

14 Ning, W. *et al.* GPS-Palm: a deep learning-based graphic presentation system for the prediction of S-palmitoylation sites in proteins. *Brief Bioinform* **22**, 1836-1847, doi:10.1093/bib/bbaa038 (2021).

15 Kostyuchenko, V. A. *et al.* The structure of barmah forest virus as revealed by cryo-electron microscopy at a 6-angstrom resolution has detailed transmembrane protein architecture and interactions. *J Virol* **85**, 9327-9333, doi:10.1128/jvi.05015-11 (2011).

16 Zhang, R. *et al.* 4.4 Å cryo-EM structure of an enveloped alphavirus Venezuelan equine encephalitis virus. *EMBO J* **30**, 3854-3863, doi:10.1038/emboj.2011.261 (2011).
